# Metabolic profiling defines glioblastoma subtypes with distinct prognoses and therapeutic vulnerabilities

**DOI:** 10.1093/neuonc/noaf294

**Published:** 2026-01-02

**Authors:** Fan Wu, Yi-Yun Yin, Di Wang, Chang-Qing Pan, You Zhai, Ming-Chen Yu, Zhi-Liang Wang, Wen-Hua Fan, Zheng Zhao, Guan-Zhang Li, Tao Jiang, Wei Zhang

**Affiliations:** Department of Molecular Neuropathology, Beijing Neurosurgical Institute, Capital Medical University, Beijing, China; Department of Neurosurgery, Beijing Tiantan Hospital, Capital Medical University, Beijing, China; Chinese Glioma Genome Atlas Network (CGGA) and Asian Glioma Genome Atlas Network (AGGA), Beijing, China; Department of Molecular Neuropathology, Beijing Neurosurgical Institute, Capital Medical University, Beijing, China; Department of Neurosurgery, Beijing Tiantan Hospital, Capital Medical University, Beijing, China; Chinese Glioma Genome Atlas Network (CGGA) and Asian Glioma Genome Atlas Network (AGGA), Beijing, China; Department of Molecular Neuropathology, Beijing Neurosurgical Institute, Capital Medical University, Beijing, China; Department of Neurosurgery, Beijing Tiantan Hospital, Capital Medical University, Beijing, China; Chinese Glioma Genome Atlas Network (CGGA) and Asian Glioma Genome Atlas Network (AGGA), Beijing, China; Department of Molecular Neuropathology, Beijing Neurosurgical Institute, Capital Medical University, Beijing, China; Department of Neurosurgery, Beijing Tiantan Hospital, Capital Medical University, Beijing, China; Chinese Glioma Genome Atlas Network (CGGA) and Asian Glioma Genome Atlas Network (AGGA), Beijing, China; Department of Molecular Neuropathology, Beijing Neurosurgical Institute, Capital Medical University, Beijing, China; Department of Neurosurgery, Beijing Tiantan Hospital, Capital Medical University, Beijing, China; Chinese Glioma Genome Atlas Network (CGGA) and Asian Glioma Genome Atlas Network (AGGA), Beijing, China; Department of Molecular Neuropathology, Beijing Neurosurgical Institute, Capital Medical University, Beijing, China; Department of Neurosurgery, Beijing Tiantan Hospital, Capital Medical University, Beijing, China; Chinese Glioma Genome Atlas Network (CGGA) and Asian Glioma Genome Atlas Network (AGGA), Beijing, China; Department of Molecular Neuropathology, Beijing Neurosurgical Institute, Capital Medical University, Beijing, China; Department of Neurosurgery, Beijing Tiantan Hospital, Capital Medical University, Beijing, China; Chinese Glioma Genome Atlas Network (CGGA) and Asian Glioma Genome Atlas Network (AGGA), Beijing, China; Department of Molecular Neuropathology, Beijing Neurosurgical Institute, Capital Medical University, Beijing, China; Department of Neurosurgery, Beijing Tiantan Hospital, Capital Medical University, Beijing, China; Chinese Glioma Genome Atlas Network (CGGA) and Asian Glioma Genome Atlas Network (AGGA), Beijing, China; Department of Molecular Neuropathology, Beijing Neurosurgical Institute, Capital Medical University, Beijing, China; Department of Neurosurgery, Beijing Tiantan Hospital, Capital Medical University, Beijing, China; Chinese Glioma Genome Atlas Network (CGGA) and Asian Glioma Genome Atlas Network (AGGA), Beijing, China; Department of Molecular Neuropathology, Beijing Neurosurgical Institute, Capital Medical University, Beijing, China; Department of Neurosurgery, Beijing Tiantan Hospital, Capital Medical University, Beijing, China; Chinese Glioma Genome Atlas Network (CGGA) and Asian Glioma Genome Atlas Network (AGGA), Beijing, China; Department of Molecular Neuropathology, Beijing Neurosurgical Institute, Capital Medical University, Beijing, China; Department of Neurosurgery, Beijing Tiantan Hospital, Capital Medical University, Beijing, China; Chinese Glioma Genome Atlas Network (CGGA) and Asian Glioma Genome Atlas Network (AGGA), Beijing, China; Department of Molecular Neuropathology, Beijing Neurosurgical Institute, Capital Medical University, Beijing, China; Department of Neurosurgery, Beijing Tiantan Hospital, Capital Medical University, Beijing, China; Chinese Glioma Genome Atlas Network (CGGA) and Asian Glioma Genome Atlas Network (AGGA), Beijing, China

**Keywords:** glioblastoma, metabolic profiling, molecular subtype, multi-omics, prognosis

## Abstract

**Background:**

Glioblastoma (GBM) is a highly aggressive brain tumor with profound metabolic heterogeneity. However, a clinically actionable classification based on metabolic gene expression remains undefined.

**Methods:**

We conducted a comprehensive multi-omics analysis of *IDH*-wildtype GBMs from three publicly available datasets. Prognostic metabolism-related genes were used to define transcriptional subtypes, which were validated in independent datasets and patient-derived cell (PDC) models. Functional assays and drug sensitivity studies were performed to explore therapeutic relevance.

**Results:**

We identified three distinct metabolic subtypes: M1, enriched for synaptic signaling and amino acid metabolism, exhibited leading-edge anatomical features; M2, characterized by mitochondrial metabolism and cell cycle activity, was associated with favorable survival; and M3, marked by hypoxia, immune activation and suppression, and broad metabolic pathway engagement, correlated with poor prognosis. These subtypes were reproducible across cohorts and faithfully recapitulated in PDC models. Metabolomic profiling confirmed distinct subtype-specific metabolic signatures. Notably, M3 cells showed high sensitivity to inhibitors targeting glycosaminoglycan degradation, nicotinamide metabolism, and retinoic acid pathways in both *in vitro* and *in vivo* models.

**Conclusion:**

Our study defines three biologically and clinically relevant metabolic subtypes of *IDH*-wildtype GBM. This classification reveals distinct metabolic programs and therapeutic vulnerabilities, providing a framework for precision metabolism-targeted strategies in GBM.

Key Points

- Multi-omics analysis identified three metabolic subtypes of *IDH*-wildtype ­glioblastoma with distinct biology, prognosis, and therapeutic targets.
- Subtype-specific vulnerabilities suggest new precision strategies for metabolism-targeted glioblastoma treatment.

Importance of the Study

Glioblastoma (GBM) remains one of the most lethal brain tumors, with limited treatment options and poor prognosis. Current molecular classifications do not adequately capture the tumor’s metabolic complexity or guide targeted therapies. This study defines three robust metabolic subtypes of *IDH*-wildtype GBM through integrative multi-omics analysis across large patient cohorts and patient-derived models. Each subtype exhibits distinct biological characteristics, prognostic outcomes, and metabolic dependencies. Importantly, the most aggressive subtype (M3) demonstrates specific vulnerabilities to metabolic pathway inhibitors, offering actionable insights for therapy. This classification provides a clinically relevant framework to stratify patients and tailor metabolism-targeted treatments, paving the way for more effective and personalized approaches in GBM management.

Glioblastoma (GBM) is the most common and aggressive primary brain tumor. Despite multimodal treatment, including maximal resection, radiotherapy, and chemotherapy, GBM typically recurs, and the median overall survival remains under two years.^1-3^ According to the 2021 World Health Organization (WHO) classification of central nervous system (CNS) tumors, GBM is defined as a diffusely infiltrative *IDH*-wildtype glioma, characterized by necrosis, microvascular proliferation (MVP), or specific molecular alterations, such as *TERT* promoter mutation, *EGFR* amplification, and the combined gain of chromosome 7 with loss of chromosome 10.^4^^,^^5^ A major obstacle to effective therapy is the pronounced heterogeneity of GBM, which spans genomic, transcriptomic, and metabolic dimensions.

Metabolic reprogramming is a hallmark of cancer,^6^^,^^7^ and in GBM, metabolic plasticity is especially prominent.^8^ GBM cells frequently exhibit aerobic glycolysis (Warburg effect), redirecting glucose metabolism to support the biosynthesis while maintaining ATP production.^9^ Additionally, these cells increase their pools of lipids, amino acids, and nucleotides through a combination of extracellular uptake, de novo synthesis, supporting growth via oxidative phosphorylation, the tricarboxylic acid (TCA) cycle, and the pentose phosphate pathway.^10^ These insights highlight the critical role of tumor metabolism in GBM biology.

Several molecular classification systems have been proposed based on transcriptional profiles,^11^^,^^12^ immune features,^13^ or pathway activity.^14^ Integrating transcriptomic and metabolomic data offers a promising approach to dissect tumor heterogeneity and define metabolic subtypes.^15-17^ However, how to stratify GBM patients based on metabolic gene expression and translate this into therapeutic insights remains an open question.

Here, we leverage multi-omics datasets to identify three robust GBM metabolic subtypes based on transcriptional profiles of metabolic genes. These subtypes show distinct metabolic gene expression signatures, genomic alterations, clinical outcomes, and sensitivities to various metabolic inhibitors, providing a potential framework for metabolism-targeted precision therapies in GBM.

## Methods

### Study Cohorts

This study included cohorts of *IDH*-wildtype GBM patients from three publicly available datasets: The Cancer Genome Atlas (TCGA), Chinese Glioma Genome Atlas (CGGA), and Clinical Proteomic Tumor Analysis Consortium (CPTAC). The TCGA cohort consisted of 139 patients with RNA sequencing data, among whom 110 had DNA methylation data, and 133 had data on copy number alterations (CNAs) and somatic mutations. The CGGA cohort included a total of 361 GBM patients, comprising 98 with microarray data and 263 with RNA-seq data. The CPTAC cohort consisted of 92 GBM patients, all of whom had RNA-seq and proteomic data, with 69 also having matched metabolomic data. A summary of the patients across all cohorts, along with their pathological features, is provided in Supplementary Table S1. Genomic, transcriptomic, and clinical data from the TCGA cohort were downloaded from the National Cancer Institute Genomic Data Commons (http://cancergenome.nih.gov).^18^ Expression and clinical data for the CGGA cohorts were obtained from the CGGA portal (http://www.cgga.org.cn).^19^ Genomic, transcriptomic, proteomic, metabolomic, and clinical data from the CPTAC cohort were accessed via the CPTAC data portal (https://proteomics.cancer.gov/programs/cptac).^20^ Transcriptomic data of PDCs from ref.^14^ are available at Synapse (accession no. syn22314624). All RNA-seq data were downloaded in FPKM format, log_2_-transformed, and standardized prior to subtype classification. Informed consent and ethical approval for all patient data used in this study were previously obtained and are documented in the respective databases.

### Metabolic Expression Subtype Classification

The TCGA cohort was used as the discovery dataset to identify metabolic subtypes of GBM. Metabolism-related genes were obtained from previously published studies,^16^^,^^21^ and genes with prognostic significance were identified using the R package “survival”. Unsupervised clustering was then performed using the consensus clustering algorithm implemented in the R package “ConsensusClusterPlus”,^22^^,^^23^ with 80% sample subsampling over 1,000 iterations and a maximum cluster number (k) set to 10. Cluster robustness was assessed both visually, through the consensus matrix heatmap, and quantitatively, using the cumulative distribution function (CDF) curves and the relative change in area under the CDF curve for each k value. To validate the identified clusters in independent cohorts, a partition around medoids (PAM) classifier was constructed using the R package “pamr”. Each GBM sample in the validation cohorts was assigned to a metabolic subtype based on the highest Pearson correlation with the centroid of each cluster and the lowest associated *P*-value.^24^ The similarity and reproducibility of metabolic subtypes between the TCGA and validation cohorts were further evaluated using the in-group proportion (IGP) statistic, implemented via the R package “clusterRepro”.^25^

### Clinical Relevance Analysis of Metabolic Expression Subtypes

Detailed analytical processes were described in Supplementary Methods.

### Biological Pathway Association and Differential Expression Analysis

See details in Supplementary Methods.

### Immune Microenvironment Analysis

To characterize the immune microenvironment across metabolic expression subtypes, multiple computational approaches were employed. The ESTIMATE algorithm^26^ was used to infer the immune and stromal content in each GBM sample based on gene expression profiles. CIBERSORT^27^^,^^28^ was applied to estimate the relative proportions of various immune cell types from bulk RNA sequencing data. In addition, single-sample gene set enrichment analysis (ssGSEA) was conducted using the R package “GSVA”^29^ to calculate enrichment scores for predefined immune-related gene signatures in each sample.

### Calculation of Metabolic Pathway Enrichment Score

To assess metabolic heterogeneity across the identified subtypes, enrichment analysis of metabolic pathways was performed. A total of 113 metabolism-related gene signatures were obtained from previously published studies.^30^ The enrichment score for each metabolic pathway in each sample was calculated using the ssGSEA method, based on transcriptomic data.

### Anatomic Enrichment Analysis

To evaluate the anatomic features among metabolic subtypes, enrichment analysis was performed with the signatures from Ivy glioblastoma atlas project (IvyGAP)^31^ and Patel et al.^32^ The scores were calculated using the ssGSEA method.

### Differential Analysis of Metabolite Profiling Data in CPTAC GBM Samples

Metabolite profiling data from 69 GBM patients were obtained from the CPTAC database^20^ and used to investigate metabolic differences among the identified subtypes. See details in Supplementary Methods.

### Somatic Driver Association Analysis

To identify oncogenic events potentially responsible for metabolic reprogramming, associations between somatic drivers, including mutations and copy number variations (CNVs), and metabolic expression subtypes were analyzed using data from the TCGA cohort. See details in Supplementary Methods.

### Cell Lines and Culturation

All PDCs used in this study were previously established and characterized.^33^ See details in Supplementary Methods.

### Compounds

Detailed information was shown in Supplementary Methods.

### In Vitro Cell Viability Assay

Detailed experimental steps were described in Supplementary Methods.

### Apoptosis Assay

Annexin V-FITC/PI (BD Pharmingen) staining was performed according to the manufacturer’s protocols. See the Supplementary Methods for details.

### NAD^+^ and Retinoic Acid Concentration Measurement

Detailed experimental steps were described in Supplementary Methods.

### β-Hexosaminidase Activity Assay

The activity of *β*-hexosaminidase was detected using beta Hexosaminidase Activity Assay Kit (Cell Biolabs). Detailed experimental steps were described in Supplementary Methods.

### Orthotopic Xenografting and Drug Treatment

The general protocol for establishing intracranial GBM models was described previously.^23^ Detailed experimental steps were described in Supplementary Methods.

### RNA Sequencing and Data Processing of PDCs

See the Supplementary Methods for details.

### Metabolite Profiling and Data Analysis of PDCs

Metabolomic analysis was performed on 17 PDC lines with available transcriptomic data. See the Supplementary Methods for details.

### Statistical Analysis

All computational and statistical analyses were performed using R software, SPSS 16.0 (IBM, Chicago, IL, USA), or GraphPad Prism 6.0 (GraphPad Inc., San Diego, CA, USA). For comparisons between two groups, the unpaired Student’s *t*-test was used for normally distributed data, while the Wilcoxon rank-sum test was applied for non-normally distributed data. For comparisons among three or more groups, one-way ANOVA was used for normally distributed variables. *P*-values were adjusted for multiple testing using the Benjamini–Hochberg method. Two-sided *P*-values < .05 were considered statistically significant.

## Results

### Metabolic Expression–Based Stratification of IDH-Wildtype GBMs

**Figure 1.**
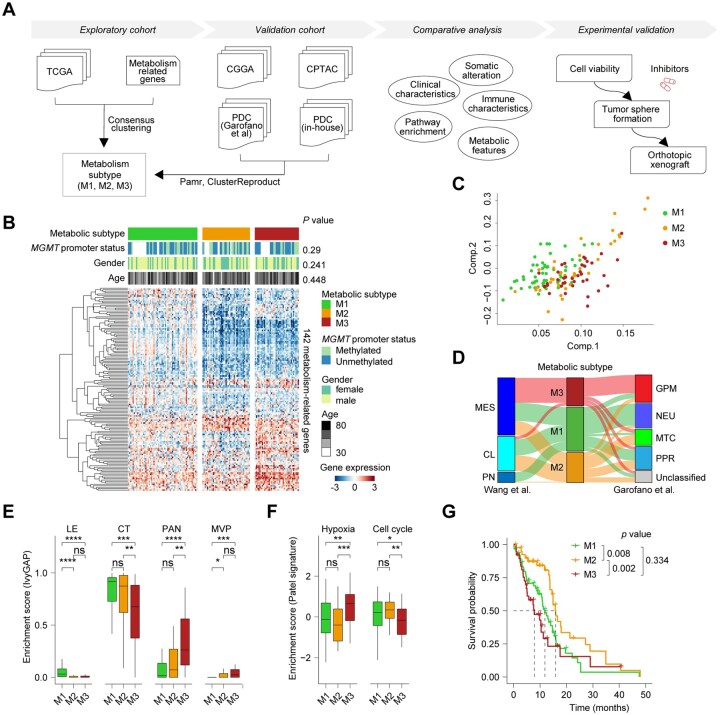
Metabolism gene profiling reveals three distinct subtypes in *IDH*-wildtype glioblastomas. (A) Flowchart illustrating the computational workflow used to classify tumor samples into metabolic expression subtypes. The TCGA cohort was used as a discovery set, while three CGGA cohorts, the CPTAC cohort, and two PDC cohorts served as validation sets. (B) Heatmap showing consensus clustering using 142 centroid genes derived from a PAM classifier in the TCGA cohort. Molecular and clinical annotations are provided for each patient, arranged by metabolic subtype. (C) Principal component analysis (PCA) of transcriptomic profiles distinguishing the three metabolic subtypes. (D) Sankey diagram comparing subtype assignments of GBM samples based on metabolic classification, Wang et al. and Luciano et al.s classification. CL, classical; GPM, glycolytic/plurimetabolic; MES, mesenchymal; MTC, mitochondrial; NEU, neuronal; PN, proneural; PPR, proliferative/progenitor. (E) Box plots showing enrichment scores of IvyGAP features among metabolic subtypes (Wilcoxon rank-sum test). *P < .05, **P < .01, ***P < .001, ****P < .0001. (F) Box plots displaying enrichment scores for hypoxia and cell cycle programs across subtypes (Wilcoxon rank-sum test). *P < .05, **P < .01, ****P < .0001. (G) Kaplan–Meier survival curves comparing overall survival (OS) across the three subtypes. *P*-values determined by log-rank test.

To uncover the metabolic heterogeneity of *IDH*-wildtype GBMs, we performed an unbiased classification based on previously reported metabolism-related genes.^16^^,^^21^ The overall workflow of the study is illustrated in Figure 1A, and the clinical characteristics of patients from all cohorts are summarized in Supplementary Table S1. We first filtered metabolism-related genes to retain those significantly associated with prognosis in the TCGA cohort using univariate survival analysis. Based on these prognostic genes, consensus clustering identified three robust metabolic subtypes, designated M1, M2, and M3, as supported by the consensus matrix and the CDF curve (Figure 1B and Supplementary Figure 1A-C). Principal component analysis (PCA) further validated the clustering, confirming distinct expression patterns across subtypes (Figure 1C). We next explored the clinical relevance of the subtypes. Chi-square tests revealed no significant associations between subtype classification and clinical features such as age, gender, or *MGMT* promoter status. However, when comparing with previously reported transcriptomic subtypes,^11^^,^^12^^,^^14^ M3 subtype was significantly associated with mesenchymal and GPM subtypes, while NEU and MTC subtypes are enriched in our M1 and M2 groups, respectively (Figure 1D and Supplementary Table S2). To further characterize the subtypes, we assessed the anatomic enrichment using the features from the IvyGAP,^31^ including leading edge (LE), cellular tumor (CT), pseudopalisading cells around necrosis (PAN), and microvascular proliferation (MVP). Subtype M1 exhibited higher LE enrichment, M2 was enriched in CT features, while M3 showed strong association with PAN and MVP (Figure 1E). In line with this, applying signatures from Patel et al.^32^ M2 subtype had higher enrichment of cell cycle, whereas M3 subtype displayed high level of hypoxia (Figure 1F). To assess the prognostic value of the metabolic subtypes, we performed Kaplan–Meier survival analysis and log-rank testing, which revealed significant differences in overall survival among the subtypes. Subtype M2 was associated with the most favorable prognosis, followed by M1 and M3 (Figure 1G, Supplementary Figure 1D). Multivariate Cox regression analysis further confirmed that M2 was an independent predictor of better survival, even after adjusting for age (Supplementary Table S3).

To evaluate the robustness of our classification, we performed consensus clustering on a randomly selected half of the TCGA samples. Again, three clusters were identified, with one cluster showing enrichment of the mesenchymal subtype and another associated with improved survival. These newly generated clusters were highly concordant with the original subtype classification (Supplementary Figure 1E-H).

We further validated the reproducibility of our classification using expression data from independent cohorts: CGGA and CPTAC. Subtype assignment in these cohorts was performed using a centroid-based classifier with Pearson correlation to the TCGA-defined subtype centroids^24^ (Supplementary Figure 2-5A). In-group proportion analysis^25^ confirmed high reproducibility across cohorts (Supplementary Table S4), and PCA again demonstrated clear separation among subtypes (Supplementary Figure 2-5B). Consistently, subtype M3 remained enriched for the mesenchymal subtype (Supplementary Figure 2-5C and Supplementary Table S5-8). Anatomical and functional features were also recapitulated in the validation cohorts: M1 showed higher LE scores, M2 retained high CT and cell cycle enrichment, and M3 was again characterized by PAN, MVP, and hypoxia (Supplementary Figure 2-5D-E). Importantly, survival analysis in CGGA cohorts confirmed that patients with M2 tumors had significantly longer overall survival compared to M1 and M3 (Supplementary Figure 2-5F-G), with multivariate Cox models again supporting the favorable prognosis associated with M2 (Supplementary Table S9-12). In contrast, the classification proposed by Wang et al. failed to effectively stratify patients by survival outcomes (Supplementary Figure 6A). Moreover, M2 tumors within the MES subtype showed better overall survival compared to M1 and M3 tumors (Supplementary Figure 6B). Collectively, these findings demonstrate that metabolic expression-based stratification defines clinically and biologically meaningful subtypes of *IDH*-wildtype GBM, highlighting the profound metabolic heterogeneity of these tumors.

### Multi-Omic Characterization of Metabolic Expression Subtypes in IDH-Wildtype GBMs

**Figure 2.**
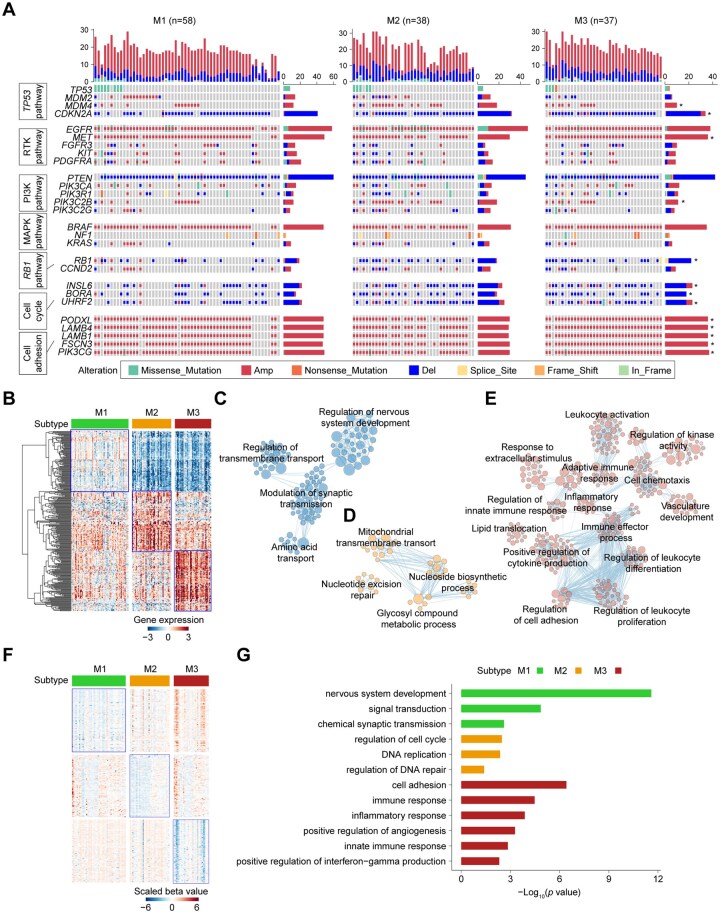
Somatic alterations, transcriptome, and DNA methylation analysis across metabolic expression subtypes in TCGA cohort. (A) Oncoprint displaying the distribution of somatic mutations and CNVs among subtypes. Fisher’s exact test used for comparisons. Highlighted pathways include TP53, RTK, PI3K, MAPK, RB1, cell cycle, and cell adhesion. *P < .05. (B) Heatmap of TCGA samples ranked by subtype using the top 100 differentially expressed genes per subtype. (C-E) GO enrichment network maps for M1 (C), M2 (D), and M3 (E) subtypes. Nodes represent enriched GO terms; edges indicate shared genes. Node size reflects the number of genes. (F) Heatmap of the top 300 differentially methylated probes across subtypes. (G) Functional annotation of hypomethylated genes in each metabolic subtype.

Accumulating evidence suggests that genomic alterations, such as *MYC* amplification and *EGFR* mutations, can drive metabolic reprogramming in GBMs.^34^^,^^35^ To identify somatic events potentially underlying the metabolic expression subtypes, we first examined the genomic alteration landscape in the TCGA cohort. There was no significant difference in overall tumor mutation burden among the subtypes (Supplementary Figure 7A). However, measures of genomic instability revealed notable differences: M3 tumors exhibited lower copy number variation burden (quantified by the number of segments) and reduced homologous recombination deficiency, but displayed higher aneuploidy scores compared to other subtypes (Supplementary Figure 7B-D), potentially driven by elevated hypoxic stress. When assessing subtype-specific associations with key GBM driver gene alterations, we found that M2 was enriched for amplifications of *MDM4* and *PIK3C2B* (Figure 2A, Supplementary Table S13). Deletions in *CDKN2A*, *RB1*, and other cell cycle-related genes (e.g., *INSL6*, *BORA*, *UHRF2*) were more frequently observed in M2 and M3 subtypes. In particular, M3 was associated with amplifications in *MET* and genes involved in cell adhesion, including *PODXL*, *LAMB4*, *LAMB1*, *FSCN3*, and *PIK3CG*.

To further understand the biological basis of these subtypes, we analyzed transcriptomic profiles using Gene Set Enrichment Analysis (GSEA). The M1 subtype was enriched for gene sets related to synaptic signaling, amino acid transport, and nervous system development (Figure 2B and C). In contrast, M2 tumors showed activation of nucleoside biosynthesis, mitochondrial metabolism, and nucleotide excision repair pathways (Figure 2D). Subtype M3 was strongly associated with immune-related responses and cell adhesion processes (Figure 2E). Similar patterns of pathway enrichment were observed in the three CGGA validation cohorts, supporting the robustness of these subtype-specific transcriptomic signatures (Supplementary Figure 7E-P).

To explore epigenetic differences across the subtypes, we conducted a DNA methylation analysis using TCGA *IDH*-wildtype GBM samples. Differentially methylated CpG sites were ranked by descending differences in beta values to identify the most subtype-specific loci (Figure 2F). We annotated hypomethylated genes in each subtype using Gene Ontology (GO) analysis, revealing consistent functional associations with transcriptomic data. In M1, hypomethylated genes were enriched in pathways related to nervous system development and chemical synaptic transmission. The M2 subtype showed epigenetic activation of genes involved in cell cycle progression, DNA replication, and DNA repair. In contrast, M3 tumors exhibited hypomethylation of genes related to cell adhesion, immune response, and angiogenesis **(**Figure 2G).

### Metabolic Expression Subtypes Exhibit Distinct Molecular and Immune Microenvironment Features

**Figure 3.**
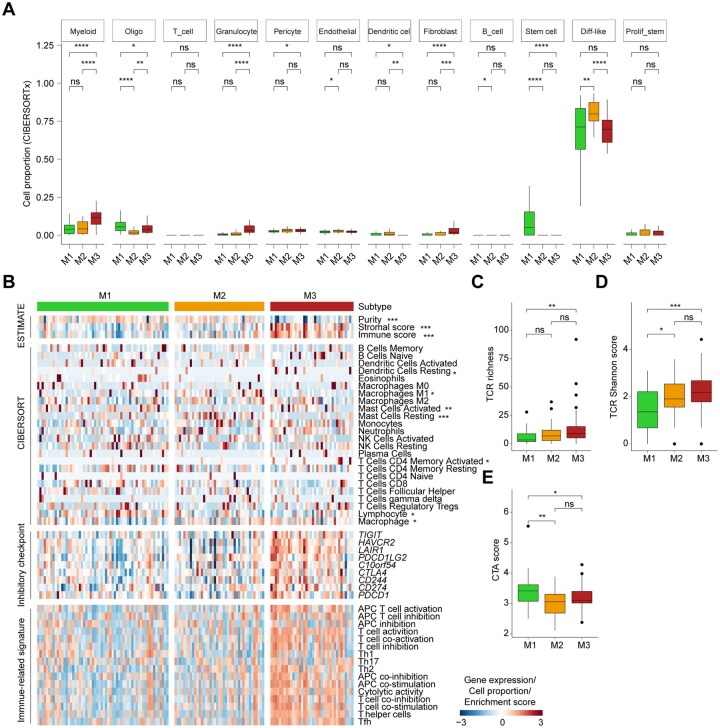
Immune infiltration analysis reveals high variability across the metabolic subtypes. (A) Box plots showing cell composition across subtypes using CIBERSORTx deconvolution (Wilcoxon rank-sum test). *P < .05, **P < .01, ***P < .001, ****P < .0001. (B) Heatmap comparing immune-related features among subtypes (ANOVA test). Immune, stromal, and purity scores from ESTIMATE; immune cell fractions from CIBERSORT; immune signatures from ssGSVA. (C-E) Box plots showing differences in TCR richness, Shannon diversity, and CTA scores (Wilcoxon rank-sum test). *P < .05, **P < .01, ***P < .001; ns, not significant.

Given the significant enrichment of immune-related pathways in our previous analyses, we next characterized the immune infiltration patterns associated with the metabolic expression subtypes using multiple established computational tools. We first assessed the cellular composition of each subtype through transcriptional deconvolution using CIBERSORTx.^36^ The M1 subtype, previously associated with LE features, was enriched in oligodendrocytes and stem-like tumor cells. In contrast, the M2 subtype, linked to CT characteristics, exhibited a higher abundance of differentiated-like tumor cells. Notably, the M3 subtype showed elevated levels of myeloid cells, granulocytes, and fibroblasts (Figure 3A). Using the ESTIMATE algorithm,^26^ we found that M3 tumors had significantly higher immune and stromal scores but lower tumor purity, consistent with a more complex and infiltrated tumor microenvironment (Figure 3B). Further immune deconvolution with the CIBERSORT algorithm^27^^,^^28^ revealed distinct immune cell distributions across subtypes. The M1 subtype exhibited higher proportions of lymphocytes, while M3 was enriched in macrophages. The M2 subtype showed a relatively higher abundance of M1 macrophages and resting mast cells, but fewer activated mast cells (Figure 3B, Supplementary Table S14). We also evaluated the expression of key immune checkpoint genes, which are involved in immune evasion mechanisms of cancer cells.^37^^,^^38^ The M3 subtype displayed significantly elevated expression of multiple inhibitory checkpoints (Figure 3B), indicating a highly immunosuppressive microenvironment. To further dissect immune functionality, we performed single-sample gene set enrichment analysis (ssGSEA)^29^ to quantify immune cell types and functional pathways. Interestingly, M3 tumors were enriched for signatures of both immune suppression and immune activation, including cytolytic activity, antigen-presenting cell (APC) regulation, and T cell activation/inhibition processes (Figure 3B), suggesting a complex and multifaceted immune landscape. These findings were validated in the CGGA and CPTAC cohorts (Supplementary Figure 8A-D). In addition, M3 tumors showed elevated protein levels of both inhibitory checkpoint molecules (e.g., *HAVCR2*, *LAIR1*, *CD274*, *VSIR*) and effector molecules (e.g., *GZMA*, *PRF1*) (Supplementary Figure 8D), reinforcing the coexistence of immune activation and suppression in this subtype.

Given that T cell receptor (TCR) diversity can reflect antigen-specific adaptive immune responses,^39^ we examined TCR repertoires from RNA-seq data. M3 tumors exhibited greater TCR diversity compared to other subtypes (Figure 3C and D), consistent with an active yet dysregulated immune response. However, despite the higher expression of cancer-testis antigens (CTAs) in the M1 subtype (Figure 3E), this did not correlate with immune activation, suggesting that CTA expression alone is insufficient to infer antitumor immune engagement.

### Metabolic Expression Subtypes Exhibit Distinct Metabolic Characteristics

**Figure 4.**
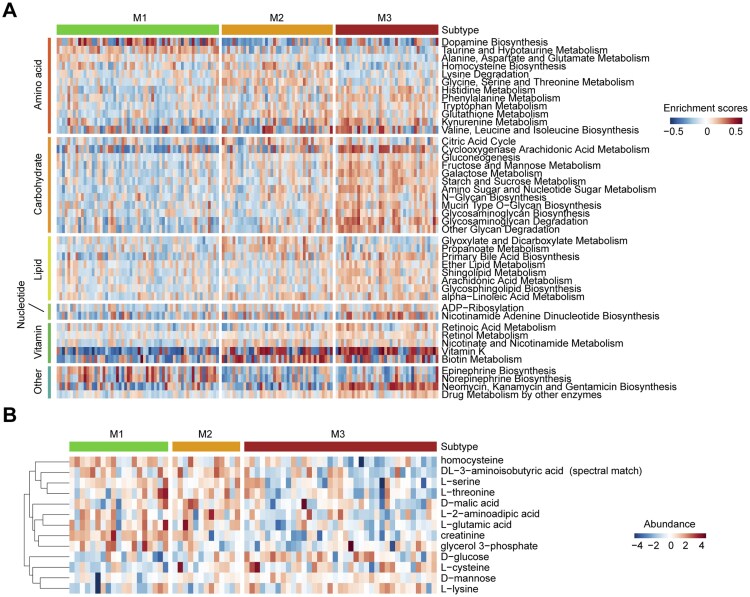
Metabolic subtypes show distinct metabolic features. (A) Heatmap illustrating differential enrichment of metabolic signatures in the TCGA cohort. Signatures include amino acid, carbohydrate, lipid, nucleotide, vitamin, and other metabolic pathways. (B) Heatmap showing differential metabolite abundances across subtypes in the CPTAC cohort (ANOVA test, *P* < .05).

To investigate whether the identified subtypes correspond to distinct metabolic features, we performed gene set variation analysis (GSVA) to estimate the enrichment scores of 113 metabolism-related pathways across all samples.^30^ Differential enrichment analysis (Supplementary Table S15) revealed that the M3 subtype was enriched in a wide range of metabolic processes, including those related to amino acid, lipid, carbohydrate, vitamin, and nucleotide metabolism. In contrast, the M2 subtype showed relative enrichment in pathways such as homocysteine biosynthesis, lysine degradation, glycine/serine/threonine metabolism, the citric acid cycle, glyoxylate, and propanoate metabolism. The M1 subtype exhibited selective upregulation of amino acid metabolic pathways, including dopamine biosynthesis, taurine/hypotaurine metabolism, and alanine/aspartate/glutamate metabolism (Figure 4A). These findings were independently validated in the CGGA and CPTAC cohorts (Supplementary Figure 9A-D, Supplementary Table S15).

To further evaluate whether these transcriptional metabolic differences translated into actual metabolite abundance, we analyzed metabolomic profiling data from the CPTAC cohort. Differential metabolite analysis (Supplementary Table S16) revealed subtype-specific metabolite signatures. Consistent with the transcriptional enrichment of lysine degradation, homocysteine biosynthesis, and serine/threonine metabolism, the M2 subtype exhibited higher levels of homocysteine, serine, and threonine, along with reduced levels of L-lysine (Figure 4B).

### Metabolic Subtypes Are Recapitulated in PDC Models of GBM

**Figure 5.**
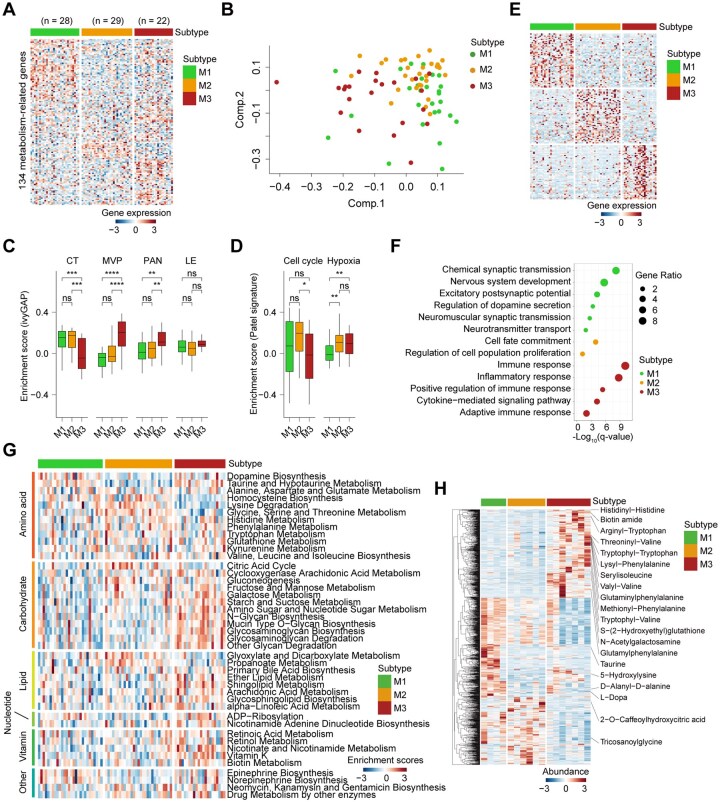
Metabolic subtypes are successfully applied to patient-derived cell (PDC) model of GBM. (A) Heatmap of consensus clustering using 134 centroid genes from the PAM classifier in the Garofano et al. PDC cohort. (B) PCA of transcriptomic data in PDCs distinguishing the three metabolic subtypes. (C-D) Box plots showing enrichment scores of IvyGAP features, hypoxia, and cell cycle programs across PDC subtypes (Wilcoxon rank-sum test). *P < .05, **P < .01, ***P < .001, ****P < .0001. (E) Heatmap of PDC samples ranked by subtype using the top 100 differentially expressed genes per subtype. (F) GO enrichment analysis of biological processes in each subtype. (G) Heatmap of differential enrichment scores for metabolic pathways in the PDC cohort. (H) Heatmap showing differentially abundant metabolites from PDC cell lines (ANOVA test).

**Figure 6.**
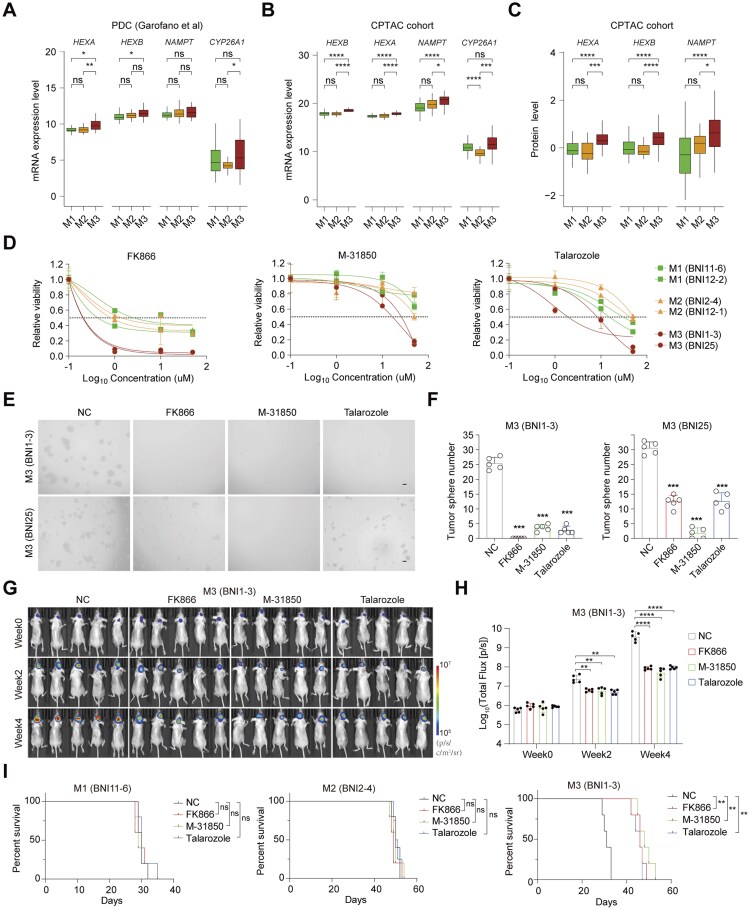
Metabolic subtypes show distinct sensitivity to metabolic inhibitors *in vitro* and *in vivo*. (A-B) Box plots showing the expression levels of *NAMPT*, *HEXA*, *HEXB*, and *CYP26A1* in PDC and CPTAC cohorts (Wilcoxon rank-sum test). *P < .05, **P < .01, ***P < .001, ****P < .0001. (C) Box plots showing the protein levels of *NAMPT*, *HEXA*, and *HEXB* in CPTAC cohort (Wilcoxon rank-sum test). *P < .05, ****P < .0001. (D) Viability curves of M1, M2, and M3 PDC cell lines treated with FK866, M-31850, and Talarozole. Data shown as mean ± s.d. n ≥ 3 per group. (E) Representative bright-field images of PDC tumor spheres under control or treatment conditions. Scale bars, 100 μm. (F) Bar plots quantifying tumor spheres between control and inhibitor-treated groups (unpaired Student’s *t*-test). ***P < .001. Data shown as mean ± s.d. (G) Representative *in vivo* bioluminescent images of nude mice bearing the intracranial xenografts treated with FK866, M-31850, and Talarozole (*n* = 5 per group). (H) Quantification of tumor growth based on *in vivo* bioluminescence in treated versus control mice (unpaired Student’s *t*-test). **P < .01, ****P < .0001. Data shown as mean ± s.d. (I) Kaplan–Meier survival curves of mice with PDC xenografts treated with metabolic inhibitors (Log-rank test). **P < .01; *n* = 5 per group.

We next investigated whether the metabolic classification of GBM could be extended to PDC models. Using transcriptomic data from Garofano et al.^14^ we applied the nearest shrunken centroids method^24^^,^^40^ to classify 79 PDCs. Remarkably, these PDCs were stratified into three distinct metabolic subtypes, consistent with those observed in GBM tissue, and exhibited corresponding transcriptional profiles and functional enrichments (Figure 5A-G). PCA confirmed distinct transcriptional patterns among the subtypes (Figure 6B). M2 PDCs were characterized by elevated levels of cell cycle and CT, while M3 PDCs showed increased activity in PAN, MVP, and hypoxia-associated signatures (Figure 5C and D). M1 PDCs exhibited upregulation of genes involved in synaptic transmission and dopamine secretion, whereas M2 PDCs were enriched in pathways related to cell proliferation. In contrast, M3 PDCs showed activation of immune-related pathways (Figure 5E and F). We further assessed the enrichment of metabolic pathways. M3 PDCs demonstrated higher activity across multiple metabolic processes, including carbohydrate, lipid, vitamin, and nucleotide metabolism. M2 PDCs displayed enrichment in pathways such as homocysteine biosynthesis, lysine degradation, glycine/serine/threonine metabolism, the citric acid cycle, glyoxylate, and propanoate metabolism. M1 PDCs selectively upregulated amino acid-related pathways, including dopamine biosynthesis, taurine/hypotaurine metabolism, and epinephrine biosynthesis (Figure 5G, Supplementary Table S15). To validate these findings, we analyzed gene expression data from 24 PDC cell lines, applying the same classification approach. The resulting subtypes exhibited consistent biological characteristics, further supporting the robustness of the metabolic classification (Supplementary Figure 10A-G). Together, these results demonstrate that the metabolic subtypes of GBM can be faithfully recapitulated in PDC models.

Next, we conducted untargeted metabolomic profiling on PDCs to compare metabolite abundance across the metabolic subtypes. Mass spectrometry analysis identified 4,143 metabolites in these samples. Principal component analysis of the metabolomic data partially recapitulated the three metabolic subtypes (Supplementary Figure 10H). Differential metabolite analysis (Supplementary Table S17) further confirmed distinct metabolic signatures among the subtypes. Consistent with pathway-level differences, M1 PDCs exhibited higher accumulation of taurine, 5-hydroxylysine, and D-alanyl-D-alanine. In contrast, M2 PDCs were enriched in metabolites such as tricosanoylglycine and 2-O-caffeoylhydroxycitric acid. M3 PDCs showed elevated levels of histidinyl-histidine, biotin amide, serylisoleucine, and various metabolites derived from tryptophan, phenylalanine, and valine (Figure 5H). These findings support the notion that metabolite abundance patterns are consistent with the distinct metabolic pathway features characterizing the three PDC subtypes.

### Metabolic Expression Subtypes Display Distinct Sensitivity to Metabolic Inhibitors

We further investigated whether the three metabolic subtypes exhibit differential sensitivity to metabolic inhibitors, given that targeting tumor metabolism has emerged as a promising therapeutic strategy.^17^ Based on the distinct metabolic characteristics of each subtype, we selected three inhibitors: M-31850, which targets glycosaminoglycan degradation via HEX inhibition^41^; FK866, a NAMPT inhibitor targeting nicotinate and nicotinamide metabolism^42^; and Talarozole, a CYP26 inhibitor that disrupts retinoic acid metabolism.^43^ These pathways showed relatively higher activity in the M3 subtype across both GBM tissue samples and PDCs (Supplementary Figure 11A-C). Consistently, expression of the respective target genes was elevated at both the mRNA and protein levels in M3 subtypes across multiple GBM and PDC cohorts (Figure 6A-C, Supplementary Figure 11D). To assess the effects of these inhibitors, we first performed *in vitro* experiments using six PDC lines representing the three subtypes: BNI11-6 and BNI12-2 (M1), BNI2-4 and BNI12-1 (M2), and BNI1-3 and BNI25 (M3). To verify on-target effects, we quantified NAD^+^ levels, *β*-hexosaminidase activity, and retinoic acid concentrations after treatment. All three biochemical readouts changed in the predicted direction, consistent with inhibition of the intended metabolic pathway (Supplementary Figure 12A-C). Cell viability assays revealed that M3 PDCs were more sensitive to all three inhibitors compared to M1 and M2 PDCs, yet displayed similar sensitivity to TMZ (Figure 6D, Supplementary Figure 12D). Consistent with this selective vulnerability, the inhibitors also induced higher levels of apoptosis in M3 PDCs (Supplementary Figure 12E-F). Similarly, sphere formation assays demonstrated consistent results, with a marked reduction in sphere number of M3 PDCs upon treatment (Figure 6E and F, Supplementary Figure 13A-B).

To further elucidate metabolic consequences, we performed GO analysis on publicly available RNA-seq datasets from glioma PDCs treated with FK866 or subjected to *HEXB* knockdown.^44^^,^^45^ FK866 led to cell-cycle inhibition and upregulation of cholesterol biosynthesis pathways, consistent with metabolic stress induced by NAD⁺ depletion. Conversely, *HEXB* knockdown caused cell-growth inhibition, reduced glycosaminoglycan metabolism and cell chemotaxis, and increased fatty acid metabolism (Supplementary Figure 12G-H). The perturbation of pathways influencing chemotactic programs suggests potential metabolic-immune coupling.

To evaluate the *in vivo* efficacy of these inhibitors, we engineered luciferase-expressing PDC lines from each subtype and implanted them into the right striatum of nude mice. After one week, mice with comparable baseline tumor burdens were randomized into control and treatment groups. *In vivo* bioluminescence imaging showed that tumor burden in mice bearing M3 PDCs (BNI1-3) was significantly reduced following treatment with the inhibitors, whereas no significant differences were observed in mice implanted with M1 or M2 PDCs (Figure 6G and H, Supplementary Figure 13C-D). Notably, survival analysis revealed that mice implanted with M3 PDCs exhibited prolonged survival upon treatment, whereas no survival benefit was observed in M1 or M2 PDC-bearing mice (Figure 6I). These findings suggest that metabolic expression-based subtyping can reveal subtype-specific metabolic vulnerabilities and may inform the development of targeted therapies for *IDH*-wildtype GBMs.

## Discussion

In this study, we successfully classified *IDH*-wildtype GBMs into three distinct metabolic subtypes. M1 subtype is enriched for synaptic signaling and amino acid metabolism. This subtype may reflect a more invasive, neuronally-associated phenotype possibly influenced by interactions with the peritumoral environment. M2 subtype is characterized by mitochondrial oxidative metabolism and cell cycle activity, and is associated with favorable patient survival. We propose that M2 represents a more metabolically efficient and proliferative but less aggressive tumor state. M3 subtype shows strong signatures of hypoxia, immune activation and suppression, and broad metabolic reprogramming, and correlates with poor prognosis. This subtype may reflect a stressed, therapy-resistant state with high microenvironmental interaction. These multi-layered profiles suggest the subtypes are not arbitrary groupings, but instead represent functionally regulated programs relevant to tumor behavior and patient outcomes.

The metabolic subtypes identified in this study might appear to be shaped by distinct genetic and epigenetic alterations. M2 tumors showed frequent *MDM4* and *PIK3C2B* amplifications which are implicated in cell cycle regulation,^46^ aligning with their proliferative, cell cycle-driven profile. Both M2 and M3 subtypes also exhibited higher rates of *CDKN2A* and *RB1* deletions, known to impact lipid and glucose metabolism.^47^ These genomic events, together with subtype-specific DNA hypomethylation, suggest that coordinated molecular changes contribute to the emergence and maintenance of distinct metabolic states. Additionally, microenvironmental cues, such as hypoxia, immune infiltration, and nutrient stress, could also shape the metabolic states. Future studies leveraging single-cell, spatial, and functional genomics approaches will be essential to dissect the regulatory networks governing metabolic state transitions and stability in GBM.

Although the subtypes identified in our study are derived from integrative, multi-omics analyses, their reproducibility across cohorts and consistent emergence in patient-derived models suggest the existence of stable, biologically relevant metabolic states. Nonetheless, given the well-documented plasticity of tumor metabolism,^48^ we acknowledge that these subtypes may represent dominant but potentially transient cellular states influenced by microenvironmental pressures, similar to the Wang et al. states previously described. The selective vulnerabilities observed in M3, in particular, support the therapeutic relevance of these states and provide a rationale for future studies aimed at dissecting their regulatory drivers and plasticity using single-cell and spatial multi-omics approaches.

Numerous studies have demonstrated that metabolic alterations within the tumor microenvironment profoundly influence immune cell function, thereby promoting tumor progression.^8^ For instance, lactic acid produced by tumor cells impairs the differentiation and activation of monocytes and T cells, while also reducing the number and cytotoxic activity of CD8⁺ T cells and natural killer (NK) cells.^49^ Similarly, increased glutamine uptake by tumor cells depletes its availability in the microenvironment, thereby impairing immune cell function.^50^ Tryptophan degradation and reduced tryptophan levels inhibit T cell activation and promote the recruitment of myeloid-derived suppressor cells (MDSCs).^51^ In this study, we performed metabolic enrichment and immune infiltration analyses and revealed distinct immune microenvironments across the metabolic subtypes. Notably, the M3 subtype, characterized by elevated tryptophan and glutamine metabolism, was also associated with increased expression of inhibitory immune checkpoint genes and enrichment of T cell suppression signatures. These findings suggest that specific metabolic preferences may shape the immune landscape of each subtype. Further investigation is warranted to elucidate how these metabolic programs reprogram the immune microenvironment and contribute to immune evasion in GBM.

Notably, the metabolic expression subtypes identified here were associated with distinct prognoses. M2 subtype, enriched for citric acid cycle activity, was associated with better prognosis. In contrast, the M3 subtype, characterized by higher activity in carbohydrate, nucleotide, and vitamin metabolism pathways, exhibited poorer survival. Similar trends were observed in prior studies.^14-16^^,^^52^ In contrast to previously reported pathway-based classification schemes,^14^ which left a substantial proportion of patients unclassified, our classification approach successfully stratifies all GBM patients across different datasets into prognostically distinct subtypes, highlighting its superior robustness and clinical utility.

We examined the prognostic relevance of 113 metabolic signature scores in GBM. Although not all signatures showed consistent correlations across the five cohorts analyzed (Supplementary Table S18), several metabolic pathways, such as fatty acid biosynthesis, pyruvate metabolism, steroid hormone metabolism, arginine biosynthesis, glycine/serine/threonine metabolism, and taurine/hypotaurine metabolism, were significantly associated with patient outcomes in at least two cohorts. Notably, glycine/serine/threonine metabolism and taurine/hypotaurine metabolism were also differentially enriched among the metabolic subtypes (Figure 4A, Supplementary Figure 9). Kaplan–Meier survival analyses revealed that these two metabolic signatures may serve as prognostic indicators (Supplementary Figure 14A-B) and could represent potential therapeutic targets in GBM.

Our metabolic classification also holds promising implications for clinical translation. While three metabolic subtypes were consistently identified across datasets, survival analyses suggest that the most clinically relevant distinction lies between M2 and the other two subtypes. This is supported by consistent survival benefit in M2 across cohorts, despite limited statistical separation between M1 and M3. These findings suggest that M1 and M3, while molecularly distinct, may represent overlapping or intermediate clinical phenotypes. Thus, a two-tiered model, distinguishing M2 from non-M2 tumors, may have value for prognostic applications, while the full three-subtype framework provides deeper biological insight.

We acknowledge that the classification presented here is shaped by the complexity of intratumoral heterogeneity in GBM, both spatial and genetic. While the three metabolic subtypes (M1-M3) reflect dominant transcriptomic and metabolic programs, they do not capture the full mosaic of cellular states present within a tumor. Single-cell analyses have consistently shown that GBMs harbor multiple co-existing phenotypes, and thus our bulk-based subtypes likely represent regional or clonal enrichments rather than uniform tumor-wide states. Additionally, the apparent stability of metabolic subtypes in patient-derived xenografts likely reflects both the selection of dominant clones at engraftment and the loss of regional microenvironmental cues, which drive metabolic plasticity *in vivo*. Thus, while our subtypes provide a meaningful framework for understanding metabolic diversity in GBM, we recognize that they represent downstream effects of a complex interplay between genetic alterations, environmental pressures, and spatial context. Future studies using spatially resolved transcriptomics and multi-region sampling will be critical to further deconvolute these relationships.

## Supplementary Material

noaf294_Supplementary_Data

## Data Availability

All data utilized in this study are publicly available from the TCGA, CGGA, and CPTAC datasets. Raw transcriptomic and untargeted metabolomic data derived from PDC cell lines have been deposited in the CGGA portal (http://www.cgga.org.cn).
