# Supplementary material for: Metabolic profiling defines glioblastoma subtypes with distinct prognoses and therapeutic vulnerabilities
Source: Neuro Oncol. 2026 Jan 2;28(4):880–94. doi: 10.1093/neuonc/noaf294 (PMC13128474; doi:10.1093/neuonc/noaf294)
Supplement: noaf294_Supplementary_Data [file noaf294_supplementary_data.zip › Supplementary figures and methods_clean.docx]

**SUPPLEMENTARY INFORMATION**

**Metabolic Profiling Defines Glioblastoma Subtypes with Distinct Prognoses and Therapeutic Vulnerabilities**

**Fan Wu et al.**

**Supplementary Figures**


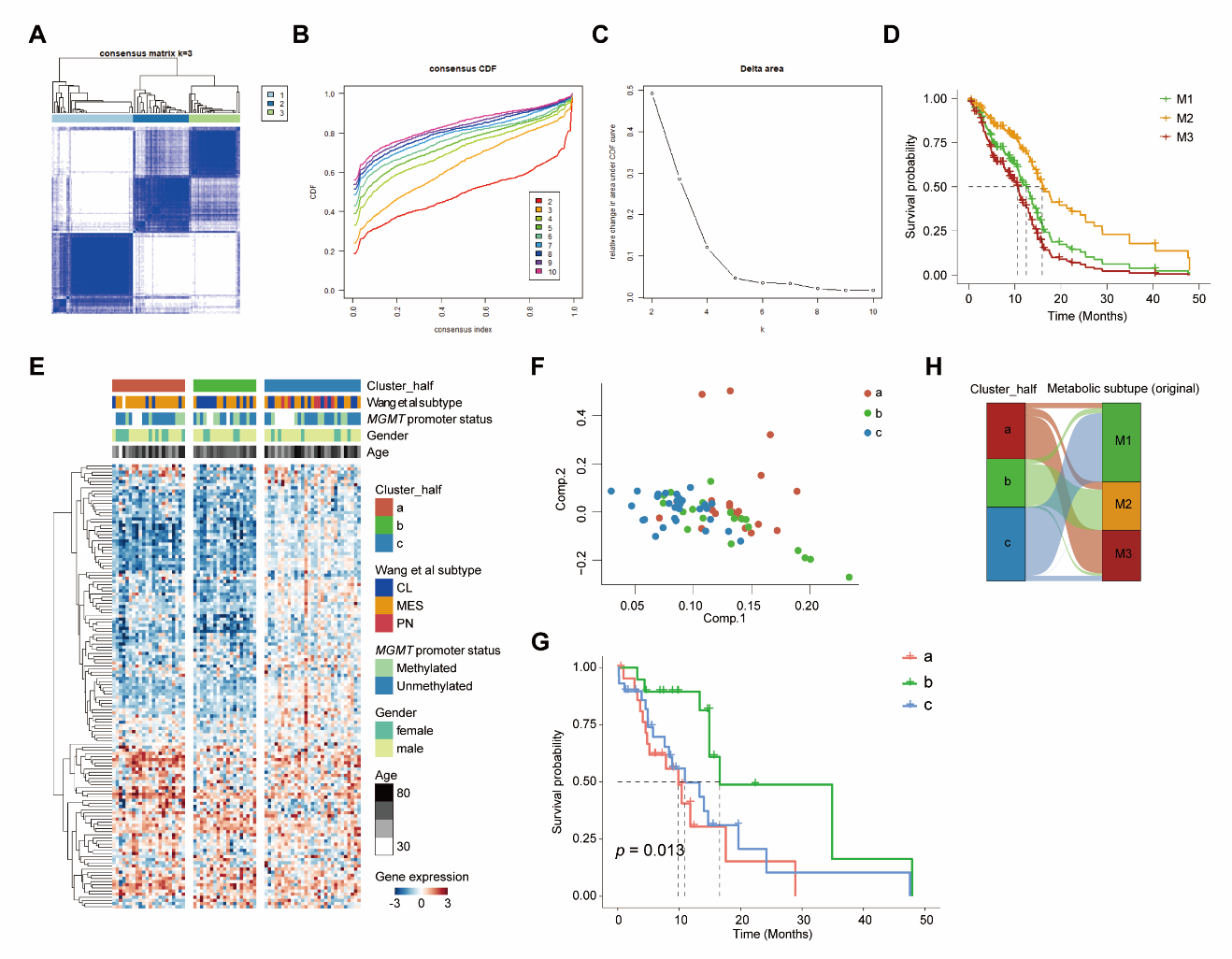


**Supplementary Figure 1. Consensus clustering based on metabolism gene expression of TCGA *IDH* wildtype GBMs.**

A. Clustering matrix for k = 3. B. CDF (cumulative distribution function) curve for k = 2 to k = 10. C. Relative change in area under CDF curve for k = 2 to k = 10. D. Survival curves of three clusters based on OS adjusted for age and gender using Cox model. E. Heatmap representing the consensus clustering after sampling in TCGA cohort. Molecular and clinical information are annotated for each patient, and patients are arranged based on the subtypes. CL, classical; MES, mesenchymal; PN, Proneural. F. Principal component analysis (PCA) of three clusters using whole transcriptome data. G. Survival analysis among three clusters based on OS. *P* value is calculated by log-rank test among subtypes. H. Sankey plot shows the concordance between clusters generated by random selection and original metabolic subtypes.


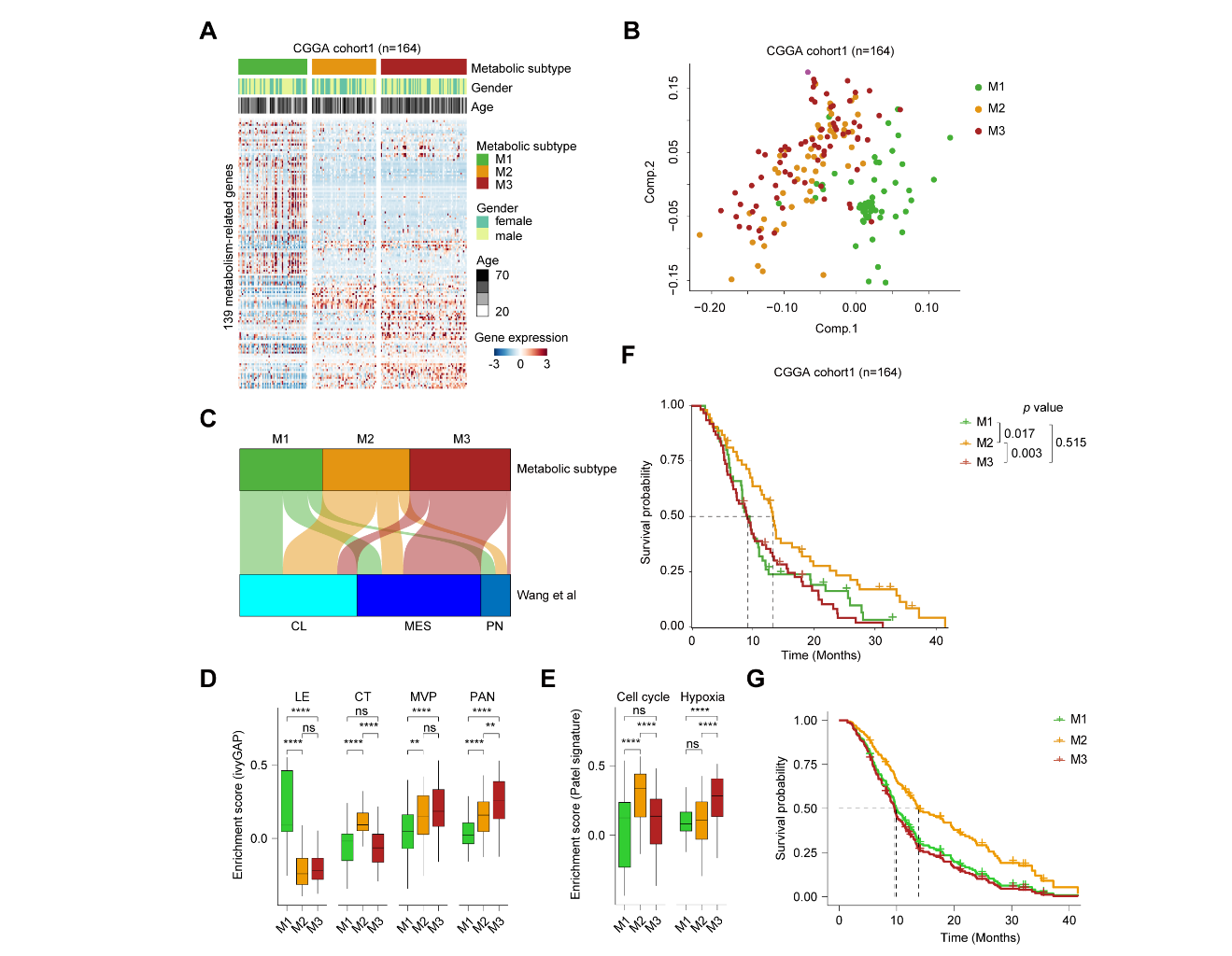


**Supplementary Figure 2. External validation of the metabolic subtypes in CGGA cohort 1.**

A. Heatmap shows the metabolic subtypes of CGGA cohort 1 predicted by a PAM classifier trained on the TCGA cohort. Samples are arranged according to the predicted subtypes. The 139 centroid genes and clinical information are displayed. B. Principal component analysis (PCA) of three subtypes using whole transcriptomic data in CGGA cohort 1. C. Sankey diagram comparing subtype assignments of GBM samples based on metabolic classification and Wang et al.’s classification. D. Box plots showing enrichment scores of IvyGAP features among metabolic subtypes (Wilcoxon rank-sum test). ***P* < 0.01, *****P* < 0.0001. E. Box plots displaying enrichment scores for hypoxia and cell cycle programs across subtypes (Wilcoxon rank-sum test). ***P* < 0.01, *****P* < 0.0001. F. The Kaplan-Meier analysis (log-rank test) of metabolic subtypes based on overall survival. G. Survival curves of three subtypes adjusted for age and gender using Cox model.


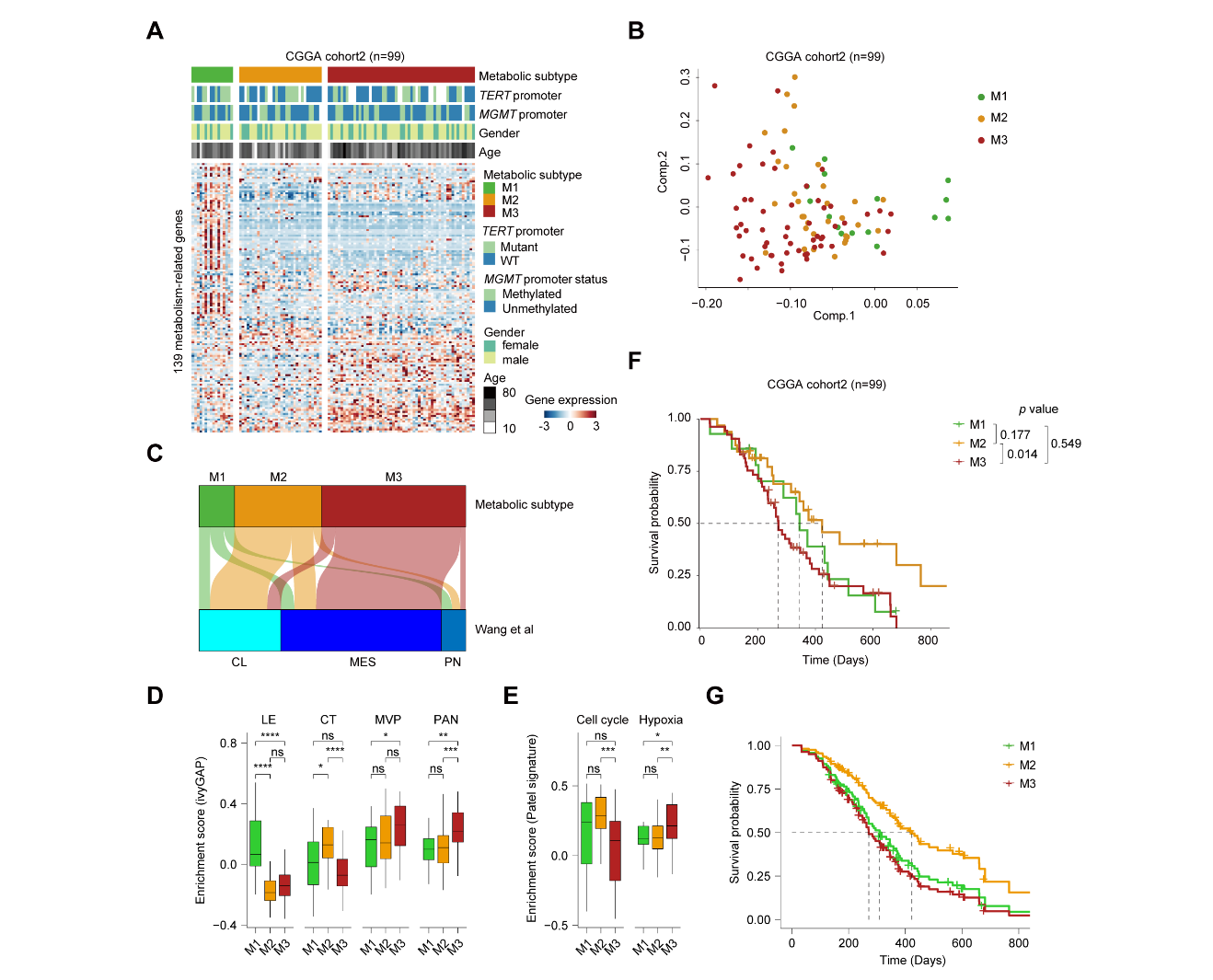


**Supplementary Figure 3. External validation of the metabolic subtypes in CGGA cohort 2**.

A. Heatmap shows the metabolic subtypes of CGGA cohort 2 predicted by a PAM classifier trained on the TCGA cohort. Samples are arranged according to the predicted subtypes. The 139 centroid genes and clinical information are displayed. B. Principal component analysis (PCA) of three subtypes using whole transcriptomic data in CGGA cohort 2. C. Sankey diagram comparing subtype assignments of GBM samples based on metabolic classification and Wang et al.’s classification. D. Box plots showing enrichment scores of IvyGAP features among metabolic subtypes (Wilcoxon rank-sum test). **P* < 0.05, ***P* < 0.01, ****P* < 0.001, *****P* < 0.0001. E. Box plots displaying enrichment scores for hypoxia and cell cycle programs across subtypes (Wilcoxon rank-sum test). **P* < 0.05, ***P* < 0.01, ****P* < 0.001. F. The Kaplan-Meier analysis (log-rank test) of metabolic subtypes based on overall survival. G. Survival curves of three subtypes adjusted for age and gender using Cox model.


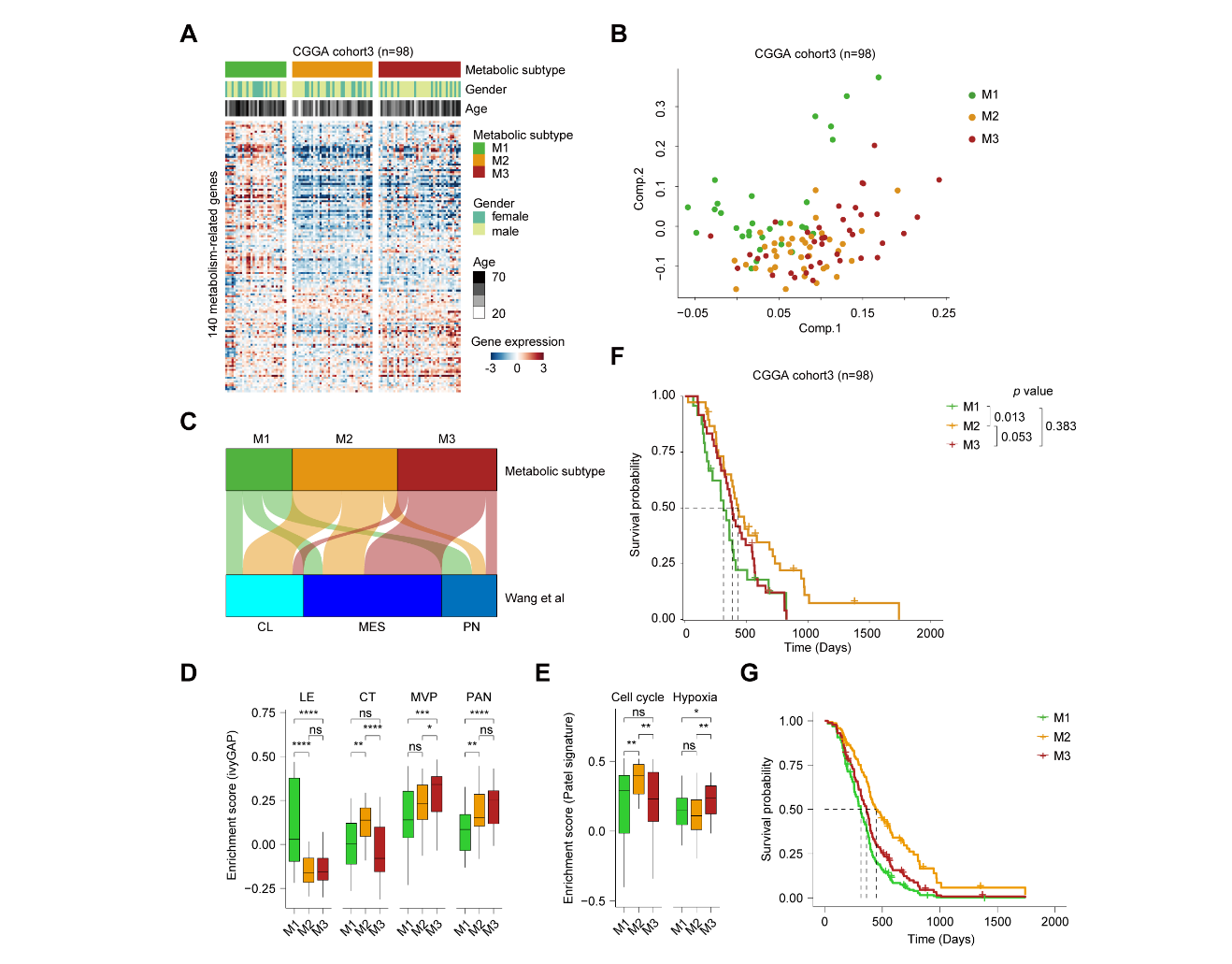


**Supplementary Figure 4. External validation of the metabolic subtypes in CGGA cohort 3.**

A. Heatmap shows the metabolic subtypes of CGGA cohort 3 predicted by a PAM classifier trained on the TCGA cohort. Samples are arranged according to the predicted subtypes. The 140 centroid genes and clinical information are displayed. B. Principal component analysis (PCA) of three subtypes using whole transcriptomic data in CGGA cohort 3. C. Sankey diagram comparing subtype assignments of GBM samples based on metabolic classification and Wang et al.’s classification. D. Box plots showing enrichment scores of IvyGAP features among metabolic subtypes (Wilcoxon rank-sum test). **P* < 0.05, ***P* < 0.01, ****P* < 0.001, *****P* < 0.0001. E. Box plots displaying enrichment scores for hypoxia and cell cycle programs across subtypes (Wilcoxon rank-sum test). **P* < 0.05, ***P* < 0.01. F. The Kaplan-Meier analysis (log-rank test) of metabolic subtypes based on overall survival. G. Survival curves of metabolic subtypes adjusted for age and gender using Cox model.


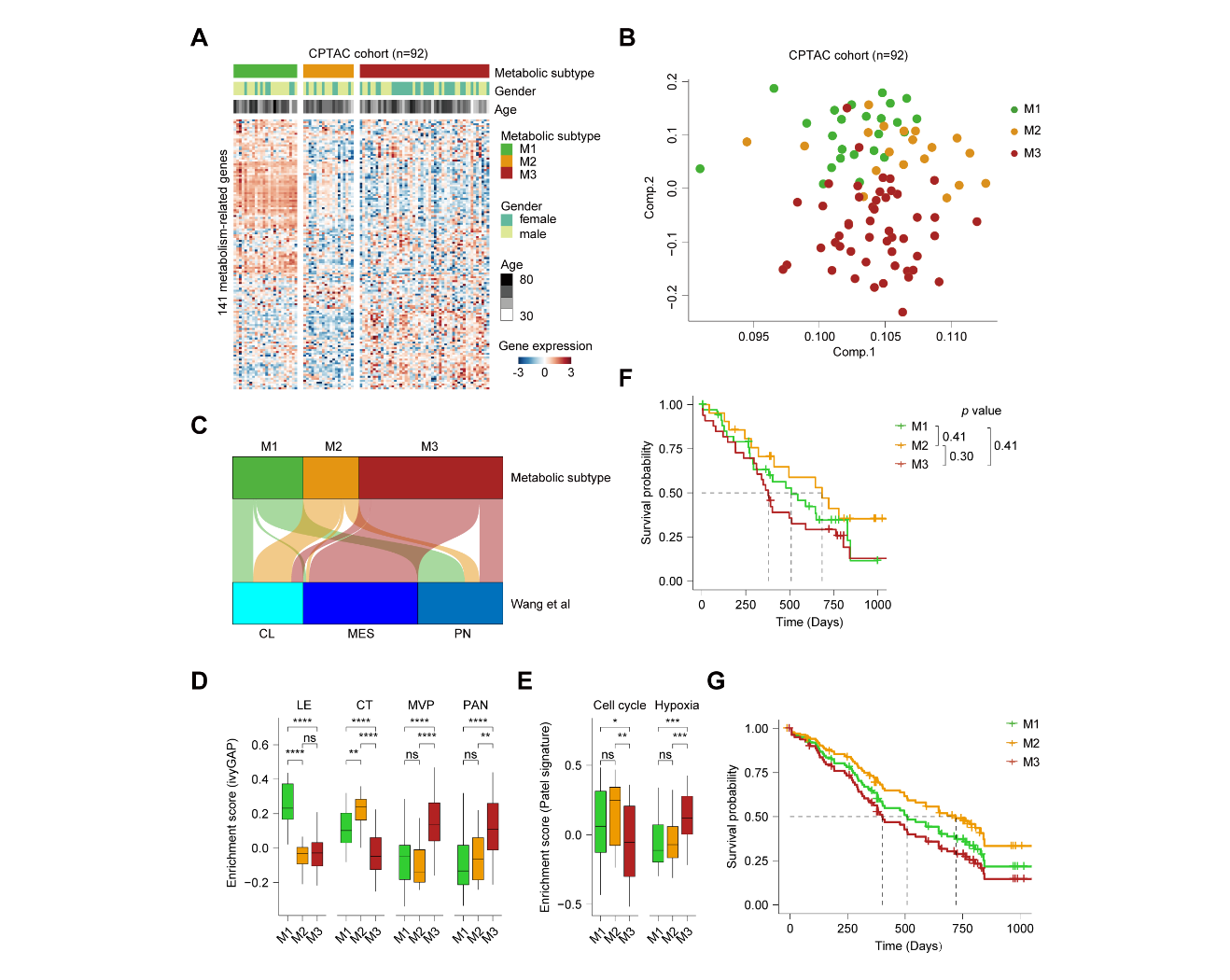


**Supplementary Figure 5. External validation of the metabolic subtypes in CPTAC cohort.**

A. Heatmap shows the metabolic subtypes of CPTAC cohort predicted by a PAM classifier trained on the TCGA cohort. Samples are arranged according to the predicted subtypes. The 141 centroid genes and clinical information are displayed. B. Principal component analysis (PCA) of three subtypes using whole transcriptomic data in CPTAC cohort. C. Sankey diagram comparing subtype assignments of GBM samples based on metabolic classification and Wang et al.’s classification. D. Box plots showing enrichment scores of IvyGAP features among metabolic subtypes (Wilcoxon rank-sum test). ***P* < 0.01, *****P* < 0.0001. E. Box plots displaying enrichment scores for hypoxia and cell cycle programs across subtypes (Wilcoxon rank-sum test). **P* < 0.05, ***P* < 0.01, ****P* < 0.001. F. The Kaplan-Meier analysis (log-rank test) of metabolic subtypes based on overall survival. G. Survival curves of metabolic subtypes adjusted for age and gender using Cox model.


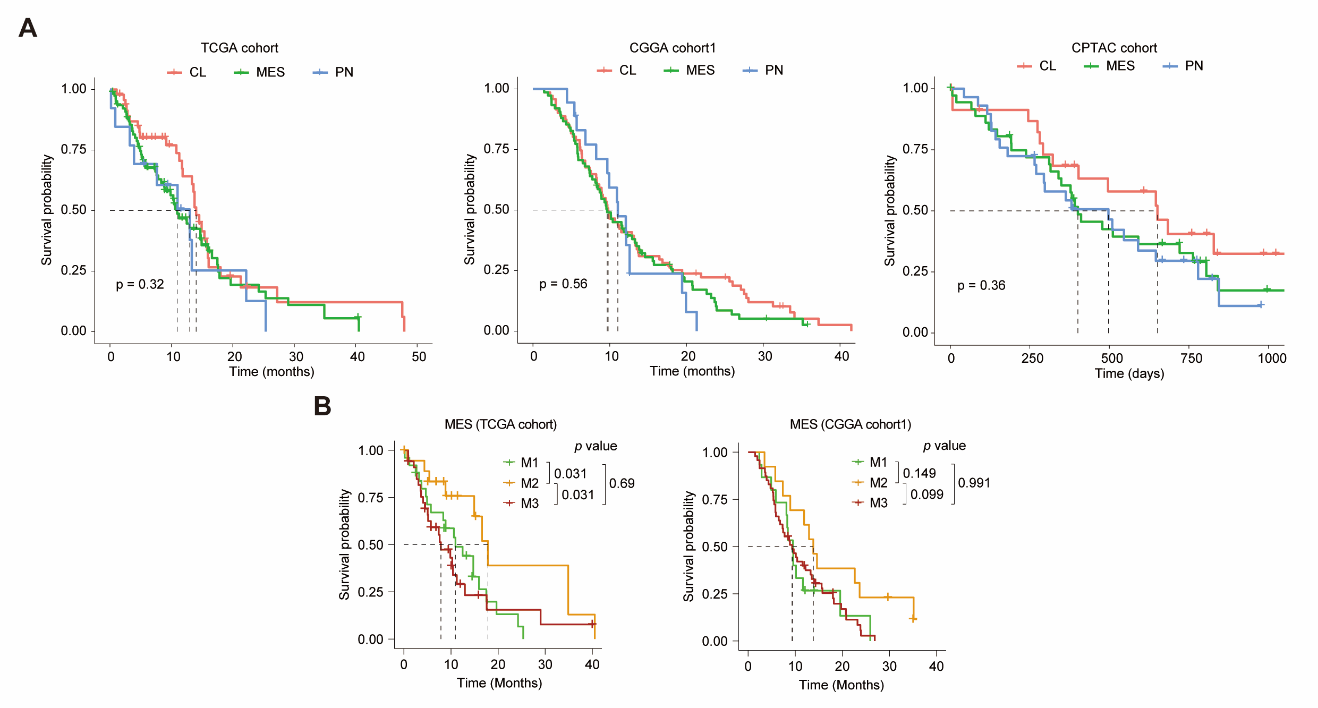


**Supplementary Figure 6. Kaplan-Meier analysis according to transcriptomic subtypes (Wang et al.) and metabolic subtypes.**

A. Kaplan-Meier survival curves comparing overall survival (OS) across the Wang et al.’s subtypes in TCGA, CGGA, and CPTAC cohorts**.** *P*-values determined by log-rank test. CL, classical; MES, mesenchymal; PN, Proneural. B. Kaplan-Meier survival curves comparing OS among metabolic subtypes in MES tumors from TCGA cohort and CGGA cohort1 (log-rank test).


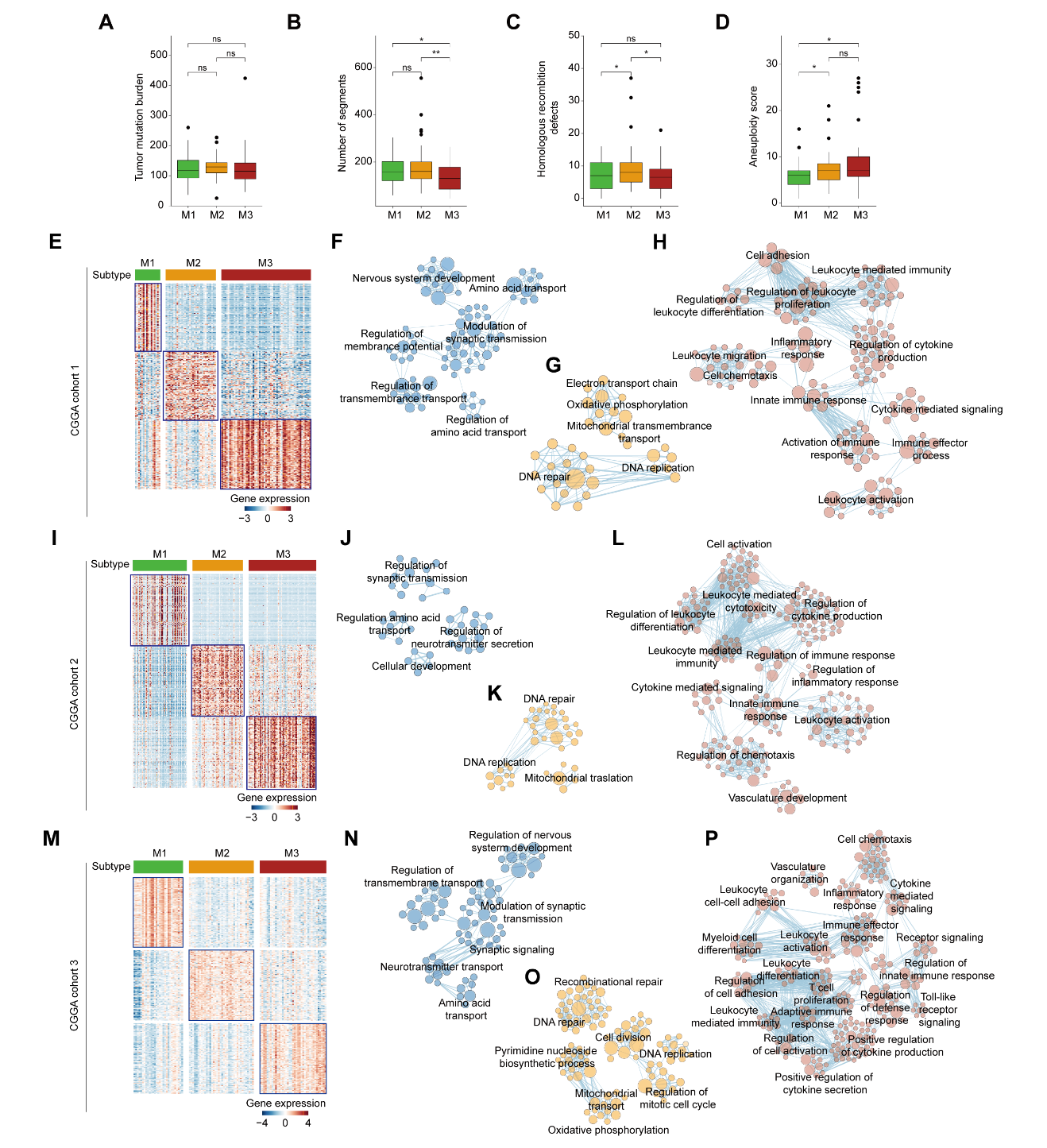


**Supplementary Figure 7. Transcriptome analysis across metabolic expression subtypes in validation cohorts.**

A-D. Box plots comparing tumor mutation burden and DNA damage-related features across metabolic subtypes (Wilcoxon rank-sum test). Center line indicates the median; error bars represent SEM. **P* < 0.05, ***P* < 0.01. E. Heatmap of CGGA cohort 1 samples ordered according to the subtypes using the top 100 differential genes of each subtype. F-H. Enrichment map network of significant GO categories in M1 (F), M2 (G), and M3 (H) subtype of CGGA cohort 1. I. Heatmap of CGGA cohort 2 samples ordered according to the subtypes using the top 100 differential genes of each subtype. J-L. Enrichment map network of significant GO categories in M1 (J), M2 (K), and M3 (L) subtype of CGGA cohort 2. M. Heatmap of CGGA cohort 3 samples ordered according to the subtypes using the top 100 differential genes of each subtype. N-P. Enrichment map network of significant GO categories in M1 (N), M2 (O), and M3 (P) subtype of CGGA cohort 3. Nodes represent GO terms, and lines their connectivity. Node size stands for proportional to the number of genes in the GO terms.


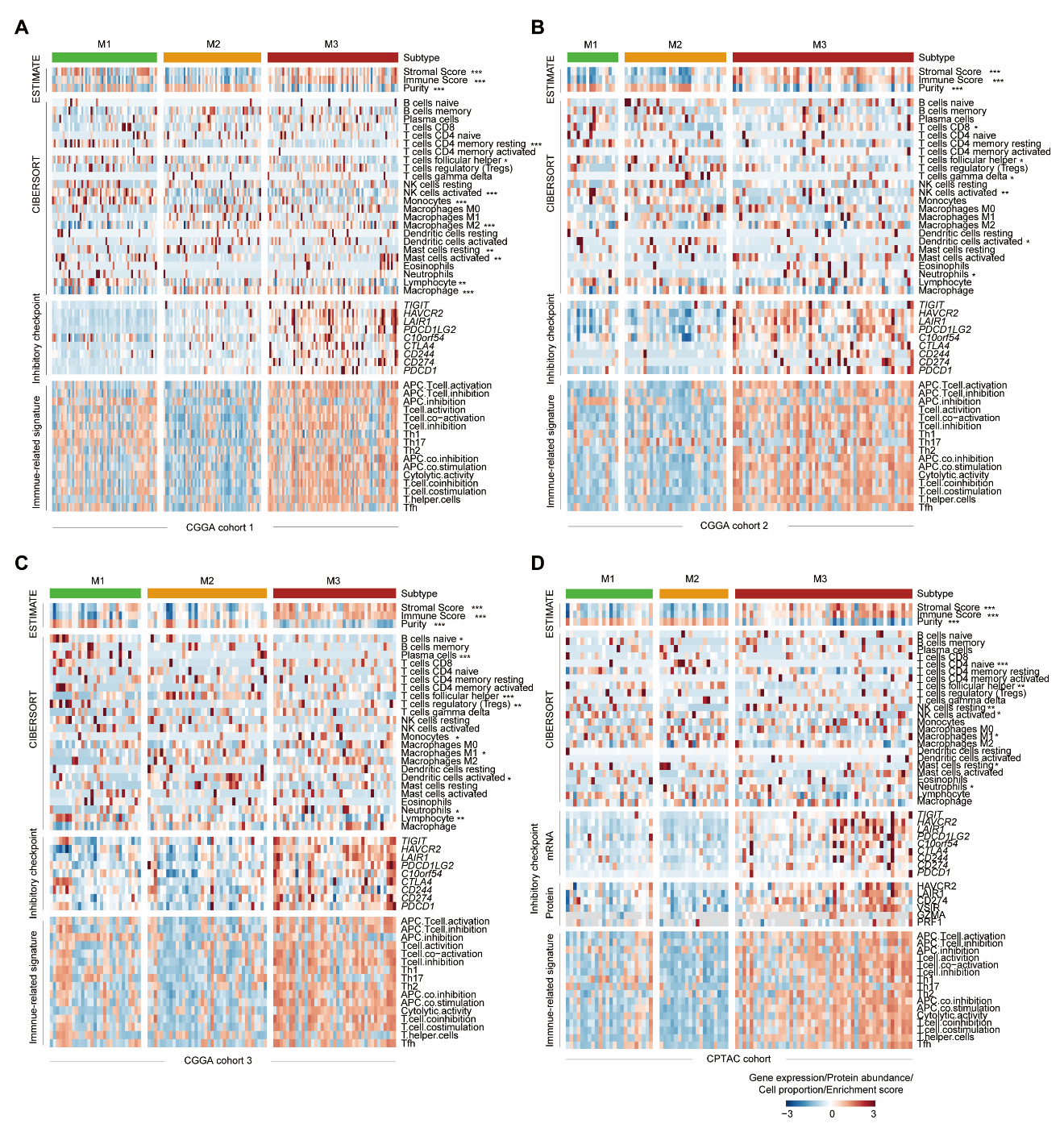


**Supplementary Figure 8. Immune infiltration analysis across the metabolic subtypes in validation cohorts.**

A-D. Heatmaps display the comparisons of immune features among metabolic subtypes (ANOVA test) in CGGA cohort 1 (A), CGGA cohort 2 (B), CGGA cohort 3 (C), and CPTAC cohort (D). Immune, stromal, and purity scores are derived from ESTIMATE. Fractions of immune cell population are calculated using CIBERCORT tool. Enrichments of immune-related signatures are evaluated using ssGSVA. **P* < 0.05, ***P* < 0.01, ****P* < 0.001.


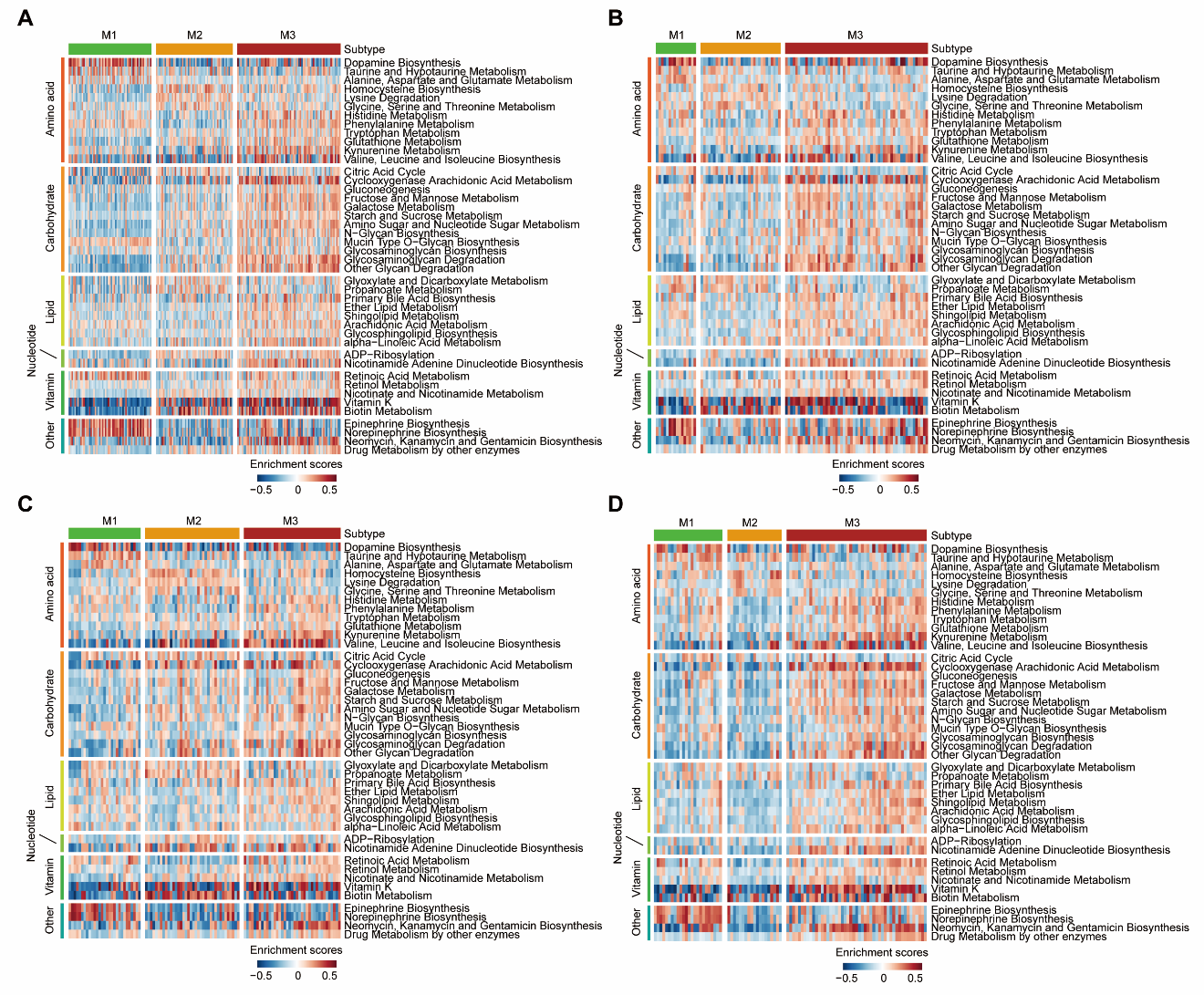


**Supplementary Figure 9. Metabolic enrichment analysis across the metabolic subtypes in validation cohorts.**

A-D. Heatmaps display the differential enrichments of metabolic-related signatures (ANOVA test) in CGGA cohort 1 (A), CGGA cohort 2 (B), CGGA cohort 3 (C), and CPTAC cohort (D). Amino acid, carbohydrate, lipid, nucleotide, vitamin, and other metabolic signatures are exhibited.


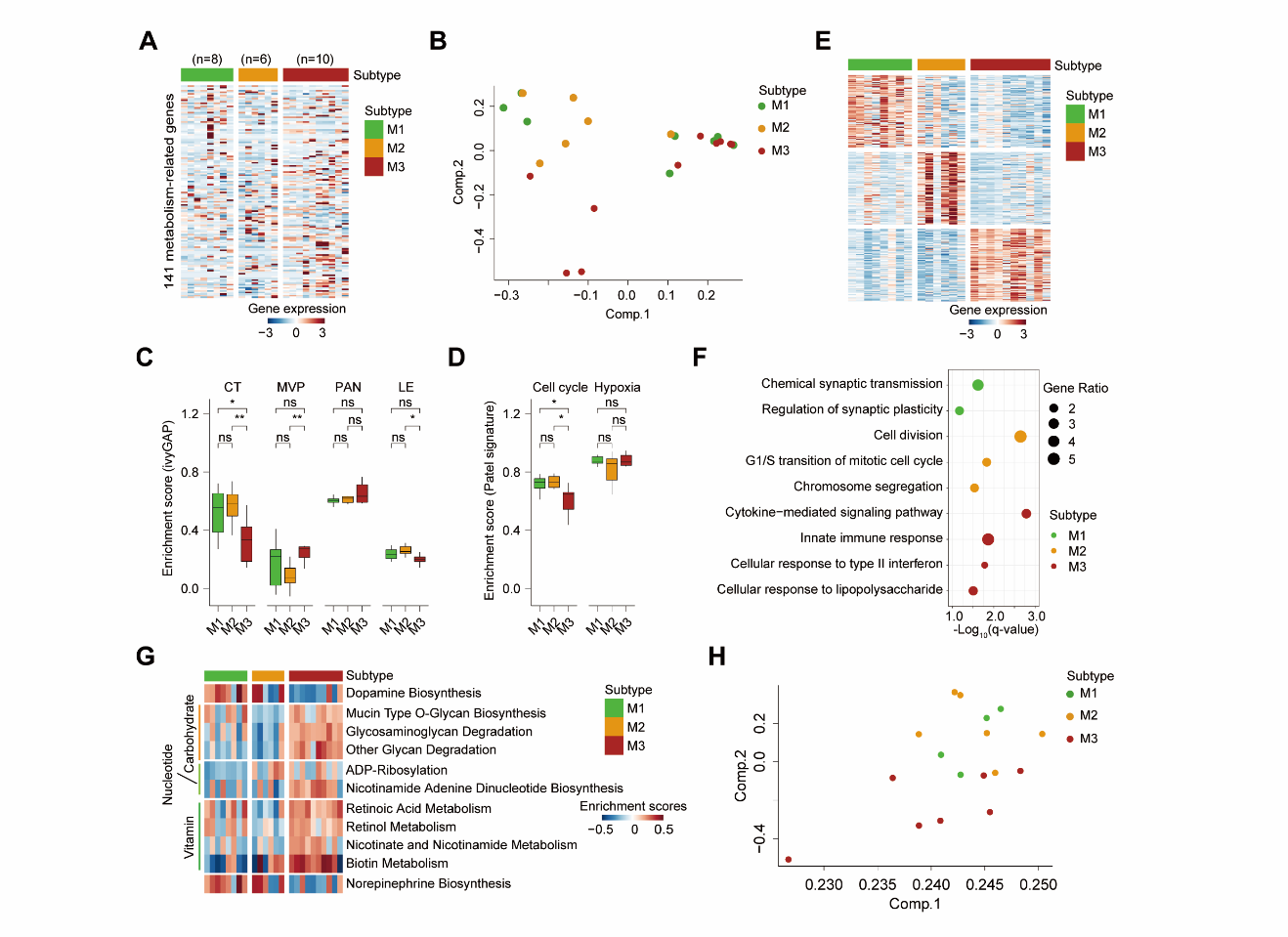


**Supplementary Figure 10. Application of metabolic classification in CGGA PDC cohort.**

A. Heatmap of consensus clustering using 141 centroid genes from the PAM classifier in the CGGA PDC cohort. B. PCA of transcriptomic data in PDCs distinguishing the three metabolic subtypes. C-D. Box plots showing enrichment scores of IvyGAP features, hypoxia, and cell cycle programs across PDC subtypes (Wilcoxon rank-sum test). **P* < 0.05, ***P* < 0.01. E. Heatmap of PDC samples ranked by subtype using the top 100 differentially expressed genes per subtype. F. GO enrichment analysis of biological processes in each subtype. G. Heatmap of differential enrichment scores for metabolic pathways in the PDC cohort. H. PCA of three metabolic subtypes using the whole metabolites.


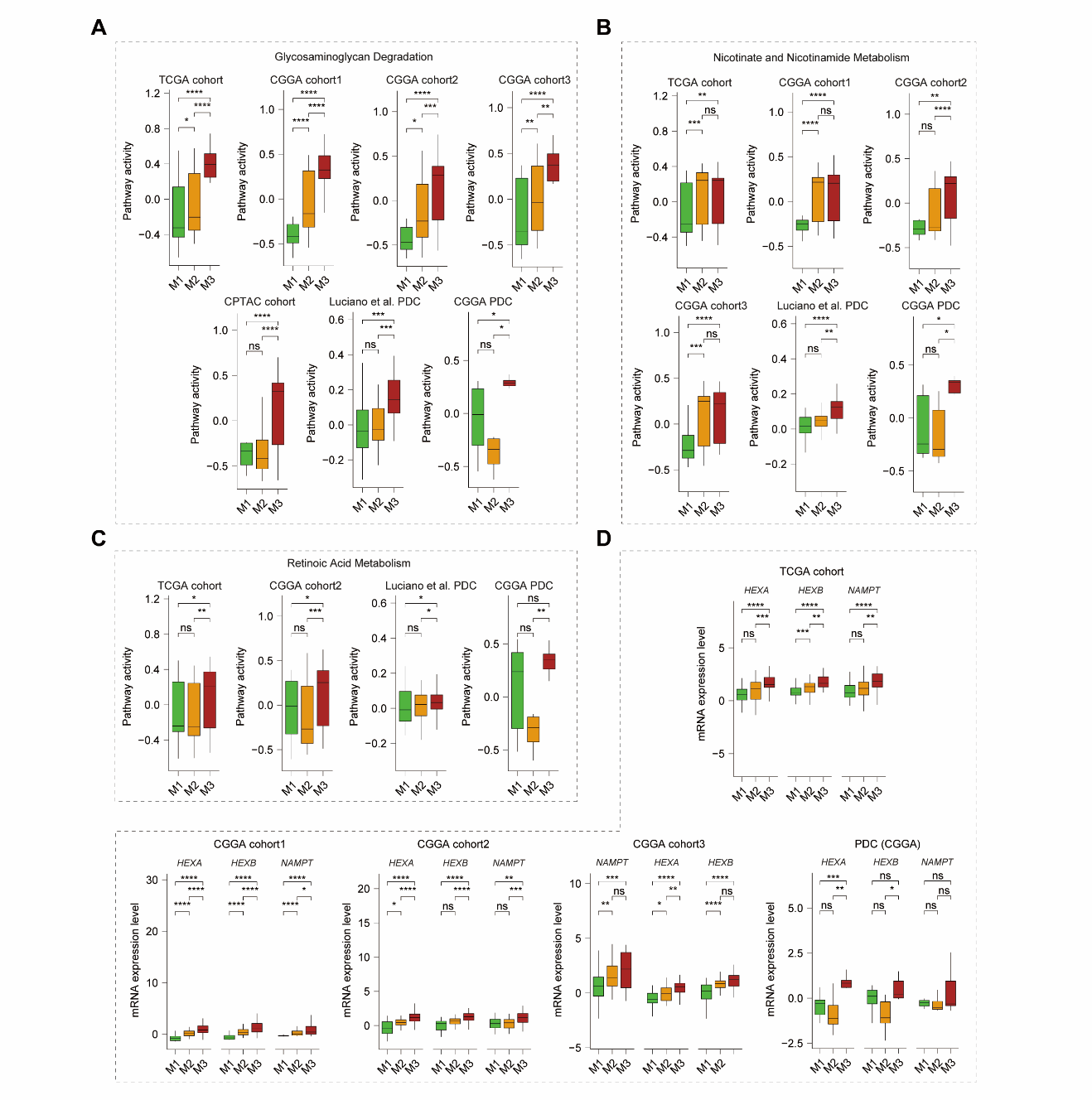


**Supplementary Figure 11. Pathway activities and expression levels of related genes across the metabolic subtypes.**

A. Pathway activities of glycosaminoglycan degradation across three metabolic subtypes in GBM tissue samples and PDCs. Wilcoxon rank-sum test is performed. **P* < 0.05, ***P* < 0.01, ****P* < 0.001, *****P* < 0.0001. B. Pathway activities of nicotinate and nicotinamide metabolism across three metabolic subtypes in GBM tissue samples and PDCs (Wilcoxon rank-sum test). **P* < 0.05, ***P* < 0.01, ****P* < 0.001, *****P* < 0.0001. ns: not significant. C. Pathway activities of retinoic acid metabolism across three metabolic subtypes in GBM tissue samples and PDCs (Wilcoxon rank-sum test). **P* < 0.05, ***P* < 0.01, ****P* < 0.001. ns: not significant. D. Gene expression in TCGA, CGGA, and PDC transcriptomic data (Wilcoxon rank-sum test). **P* < 0.05, ***P* < 0.01, ****P* < 0.001, *****P* < 0.0001. ns: not significant.


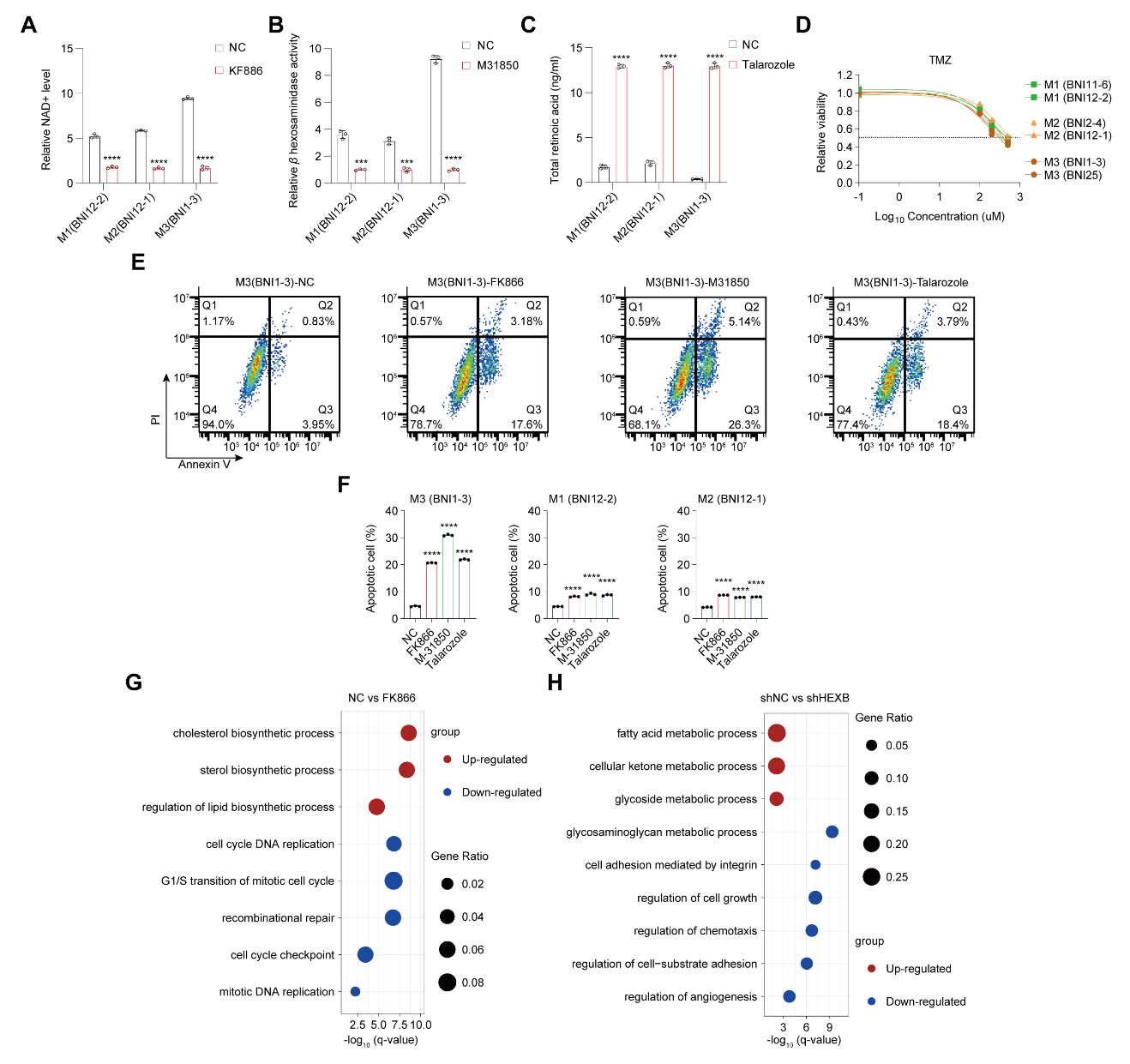


**Supplementary Figure 12. Effect of metabolic inhibitors on cell apoptosis and biological pathways.**

A. Bar plot shows the relative NAD^+^ level between control and inhibitor-treated groups (unpaired Student’s t-test). Data shown as mean ± s.d, *****P* < 0.0001. B. Bar plot shows the relative *β* hexosaminidase activity between control and inhibitor-treated groups (unpaired Student’s t-test). Data shown as mean ± s.d, ****P* < 0.001, *****P* < 0.0001. C. Bar plot shows total retinoic acid concentration between control and inhibitor-treated groups (unpaired Student’s t-test). Data shown as mean ± s.d, *****P* < 0.0001. D. Viability curves of M1, M2, and M3 PDC cell lines treated with Temozolomide (TMZ). Data shown as mean ± s.d. n ≥ 3 per group. E. Representative flow cytometry images of M3 PDC treated with FK866, M-31850, and Talarozole. F. Quantification of apoptotic cells percentage of PDCs treated with FK866, M-31850, and Talarozole (n = 3 per group, unpaired Student’s t-test). Data shown as mean ± s.d, *****P* < 0.0001. G. GO analysis on publicly available RNA-seq dataset from glioma PDCs treated with FK866. H. GO analysis on publicly available RNA-seq dataset from glioma PDCs subjected to HEXB knockdown.


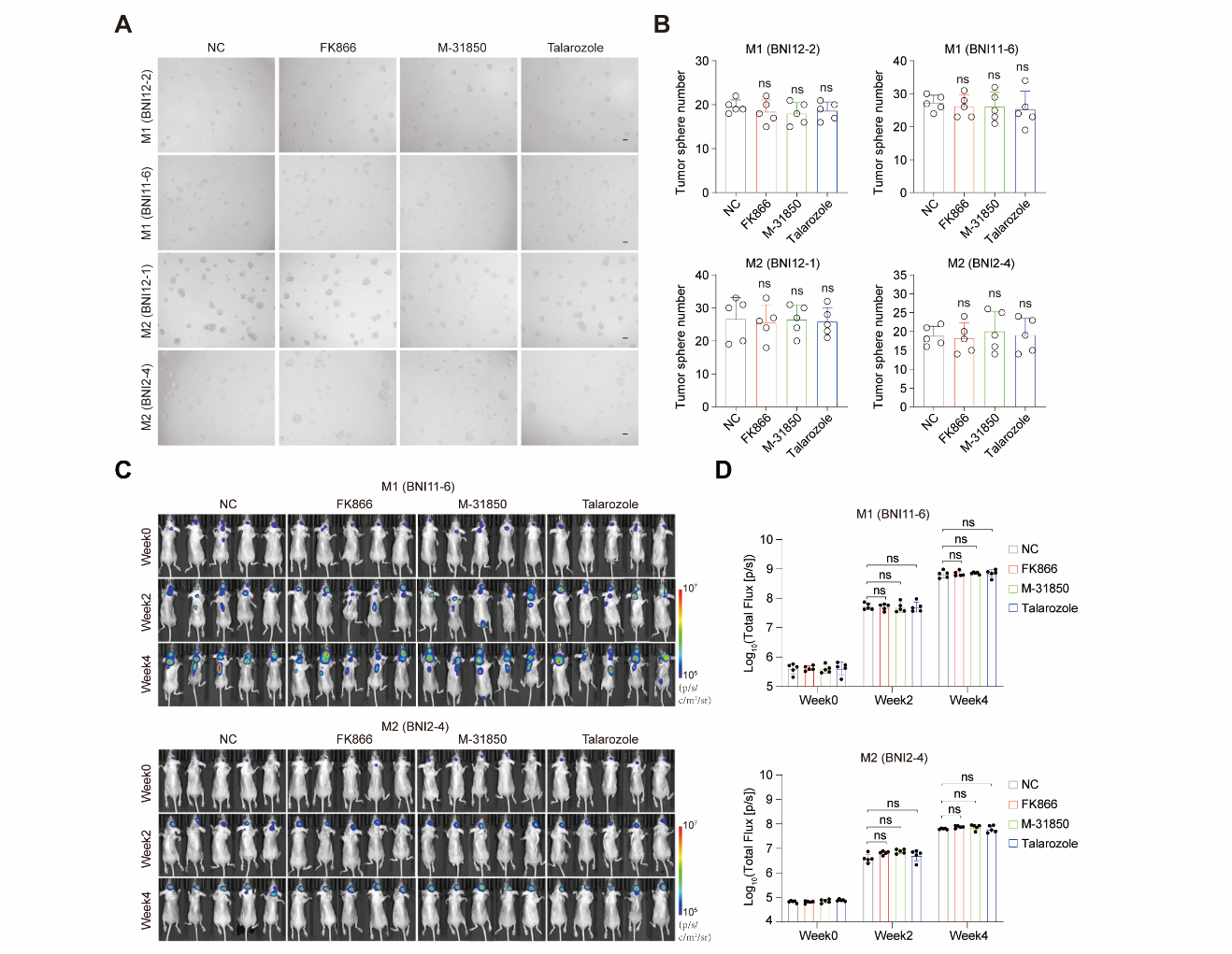


**Supplementary Figure 13. M1 and M2 PDC subtypes are not sensitive to the selected metabolic inhibitors.**

A. Representative bright-field images of M1 and M2 PDC tumor spheres under control or treatment conditions. Scale bars, 100 μm. B. Bar plots quantifying tumor spheres between control and inhibitor-treated groups (unpaired Student’s t-test). Data shown as mean ± s.d, ns: not significant. C. Representative *in* *vivo* bioluminescent images of nude mice bearing the intracranial xenografts treated with FK866, M-31850, and Talarozole (n = 5 per group). D. Quantification of tumor growth based on *in vivo* bioluminescence in treated versus control mice (unpaired Student’s t-test). Data shown as mean ± s.d, ns: not significant.


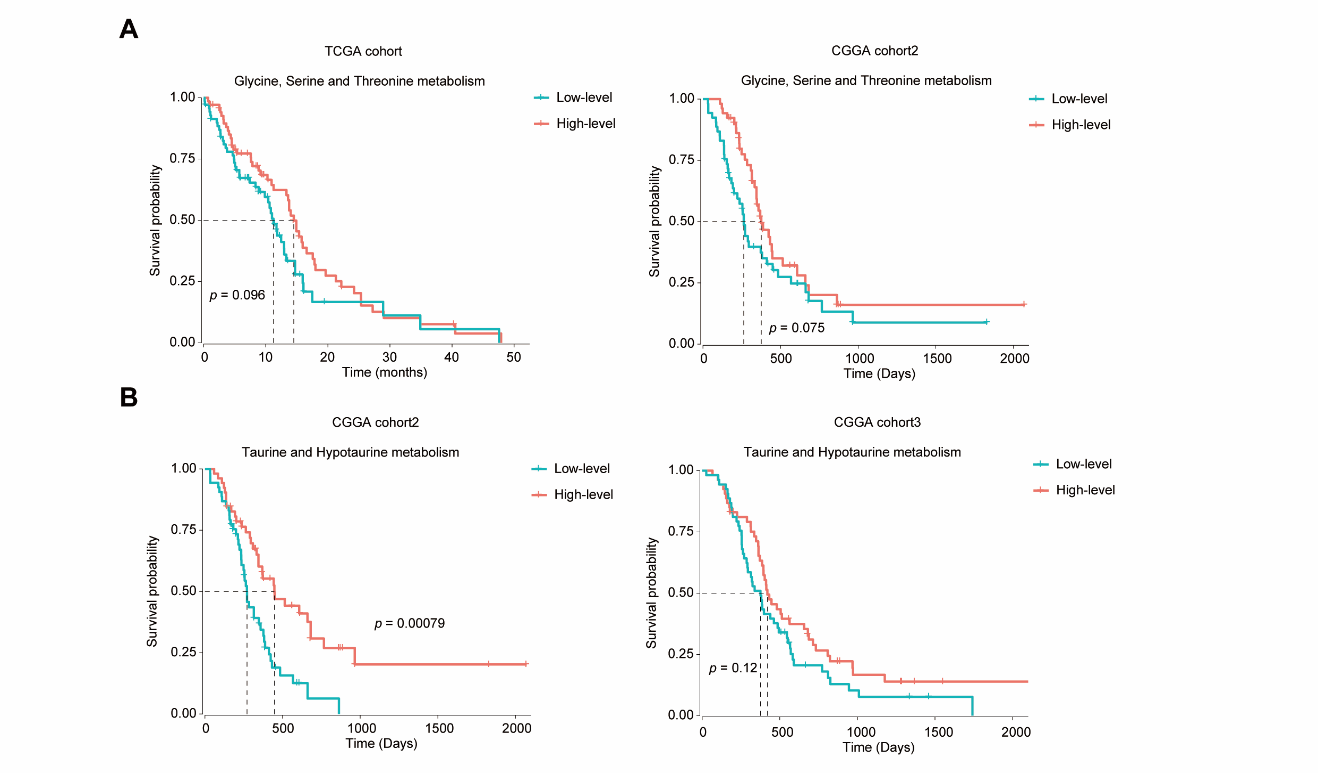


**Supplementary Figure 14. Prognostic correlation of two metabolism signatures.**

A. Kaplan-Meier analysis (log-rank test) of Glycine, Serine and Threonine metabolism signature in TCGA and CGGA cohort2. B. Kaplan-Meier analysis (log-rank test) of taurine and hypotaurine metabolism signature in CGGA cohort2 and cohort3.

**Supplementary Methods**

**Clinical relevance analysis of metabolic expression subtypes**

The associations between metabolic expression subtypes and clinical features were systematically evaluated. Overall survival analysis was performed using the R package “survival”, and Kaplan-Meier survival curves were generated. Statistical significance was assessed using the log-rank test. The chi-square test was employed to evaluate the association between metabolic subtypes and categorical molecular characteristics. For subtypes showing significant survival differences, univariable Cox proportional hazards regression analysis was conducted. Clinical covariates including age, gender, and *MGMT* promoter methylation status were incorporated into the model. Furthermore, multivariable Cox regression analysis was performed to adjust for potential confounding variables, allowing for the identification of subtype-specific prognostic effects while controlling for multiple clinical factors.

**Biological pathway association and differential expression analysis**

To investigate the biological processes underlying the metabolic expression subtypes, gene set enrichment analysis (GSEA) was performed using the GSEA software (version 3.0)^1^ with curated gene sets from the Molecular Signatures Database (MSigDB; http://www.broad.mit.edu/gsea/).^2^ Enrichment networks were visualized using Cytoscape (version 3.7)^3^ to provide an intuitive representation of the functional pathways. Differential gene expression analysis between metabolic subtypes was conducted using the DESeq2 package (version 1.26),^4^ allowing for the identification of subtype-specific transcriptional signatures.

**Differential analysis of metabolite profiling data in CPTAC GBM samples**

Metabolite profiling data from 69 GBM patients were obtained from the CPTAC database^5^ and used to investigate metabolic differences among the identified subtypes. A total of 83 annotated metabolites were included in the analysis. Differential abundance of metabolites between subtypes was assessed using the Wilcoxon rank-sum test. *P*-values were adjusted for multiple testing using the Benjamini-Hochberg method to control the false discovery rate.

**Somatic driver association analysis**

To identify oncogenic events potentially responsible for metabolic reprogramming, associations between somatic drivers, including mutations and copy number variations (CNVs), and metabolic expression subtypes were analyzed using data from the TCGA cohort. For mutation analysis, significantly mutated genes were selected, and associations with metabolic subtypes were evaluated using the fisher test based on mutation status. For CNV analysis, the copy number status of genes located within significant amplification or deletion peaks, identified using GISTIC2,^6^ was similarly assessed using the fisher test.

**Cell lines and culturation**

All patient-derived cells (PDCs) used in this study were previously established and characterized.^7^ Cells were cultured in Neurobasal medium (GIBCO) supplemented with 20 ng/mL basic fibroblast growth factor (bFGF; PeproTech), 20 ng/mL epidermal growth factor (EGF; PeproTech), 2% B27 supplement (GIBCO), and 10 µg/mL heparin (Sigma). Cultures were maintained in a humidified incubator at 37 °C with 5% CO₂. The identity of each cell line was verified by short tandem repeat (STR) profiling.

**Compounds**

The nicotinamide phosphoribosyltransferase (*NAMPT*) inhibitor FK866, the β-hexosaminidase (Hex) inhibitor M-31850, and retinoic acid metabolism blocking agent Talarozole were purchased from MedChemExpress company.

***In vitro* cell viability assay**

Cells were seeded in laminin-coated 96-well plates (Invitrogen) and allowed to adhere overnight. Optimal seeding densities were determined for each cell line to ensure approximately 85% confluence at the end of the assay. The following day, the medium was replaced with 100 µL of fresh medium containing the indicated concentrations of various inhibitors or Temozolomide. After 72 hours of treatment, Cell Counting Kit-8 (CCK-8) reagent was added to each well and incubated for 1 hour at 37 °C. Absorbance was measured at 450 nm using a microplate reader. The half-maximal inhibitory concentration (IC₅₀) was calculated by fitting a four-parameter logistic curve.

For the tumor sphere formation assay, 200 cells were seeded into each well of a 96-well plate in 100 µL of fresh medium containing 10 µM of the indicated inhibitors. After 10 days of culture, wells were imaged, and tumor spheres with a diameter greater than 100 µm were counted.

**Apoptosis assay**

5 × 10⁵ cells were seeded into 6-well plates and incubated with different inhibitors for 48 h. Annexin V-FITC/PI (BD Pharmingen) staining was performed according to the manufacturer’s protocols. The apoptosis rate was analyzed using Accuri C6 flow cytometer (BD Pharmingen).

**NAD^+^ and retinoic acid concentration measurement**

The NAD^+^ concentration was determined using a NAD^+^/NADH quantification colorimetric kit (MCE) according to the manufacturer’s protocol. Absorbance at 450 nm was measured with a microplate reader. The Retinoic acid (RA) concentration was measured with Human Retinoic Acid ELISA kit (MyBioSource). PDC cells were treated with Talarozole and cell lysis was prepared to measure the RA level by following the instruction. Optical Density (OD) at 450 nm was was measured with a microplate reader in 15 minutes.

***β*-Hexosaminidase activity assay**

The activity of *β*-hexosaminidase was detected using beta Hexosaminidase Activity Assay Kit (Cell Biolabs). PDC cells were treated with Hex inhibitor (M-31850) and cell lysis was prepared to measure the activity by following the instruction. Absorbance at 450 nm was measured with a microplate reader.

**Orthotopic xenografting and drug treatment**

Briefly, 5 × 10⁵ patient-derived glioblastoma cells (PDCs) stably expressing a luciferase reporter were stereotactically injected into the right striatum of 6-week-old BALB/c nude mice. One week post-implantation, tumor engraftment was confirmed by bioluminescence imaging, and mice were randomly assigned to treatment groups: FK866 (10 mg/kg, intraperitoneal), M-31850 (0.2 mg/kg, intraperitoneal), Talarozole (2.5 mg/kg, intraperitoneal), or vehicle control. Drug treatments were administered once daily for five consecutive days, followed by two days off (5-on, 2-off cycle). Tumor progression was monitored weekly by imaging, and survival was recorded. Mice exhibiting severe neurological symptoms, such as dome-shaped head or hemiparesis, were humanely euthanized. All animal procedures were approved by the Animal Care and Use Committee of Tiantan Hospital (Protocol No. VST20240901-1), and conducted in accordance with institutional and national ethical guidelines.

**RNA sequencing and data processing of PDCs**

Total RNA was extracted from 24 patient-derived cell (PDC) lines using the RNeasy Mini Kit (Qiagen), following the manufacturer’s instructions. RNA libraries were prepared according to the standard Illumina protocol and sequenced on the Illumina HiSeq platform. The resulting FASTQ files were aligned to the human reference genome (GENCODE v19, hg19) using STAR aligner (version 2.5.2b).^8^ Gene-level read counts were quantified using RSEM (version 1.2.31).^9^ Expression values were normalized as Fragments Per Kilobase of exon model per Million mapped fragments (FPKM), and an expression matrix was generated by merging the FPKM values across all PDC samples.

**Metabolites profiling and data analysis of PDCs**

Metabolomic analysis was performed on 17 patient-derived cell (PDC) lines with available transcriptomic data. Metabolites were extracted using methanol: water (4:1, v/v) containing 2-chloro-L-phenylalanine (0.3 mg/mL in methanol) as an internal standard. Cells were disrupted using an ultrasonic homogenizer, and the extracts were centrifuged at 4 °C. The supernatant was dried using a vacuum freeze concentrator, then reconstituted in methanol:water, vortexed, centrifuged, and filtered through 0.22 µm membranes. Filtrates were transferred to vials for LC-MS and GC-MS analysis.

For GC-MS, derivatized samples were analyzed using an Agilent 7890B gas chromatograph coupled with a 5977A mass spectrometer. A DB-5MS capillary column (30 m × 0.25 mm × 0.25 µm) was used with an injector temperature of 260 °C. Data were acquired in full scan mode (m/z 50–500), converted using AnalysisBaseFileConverter, and processed using MS-DIAL software. Metabolite identification was performed against the LUG database (Lumingbio).

For LC-MS, samples were analyzed on an ACQUITY UPLC I-Class system coupled with a VION IMS QTOF mass spectrometer (Waters Corporation) using an ACQUITY UPLC BEH C18 column (1.7 µm, 2.1 × 100 mm) in both positive and negative ESI modes. Data were acquired in full-scan mode with MSE acquisition and analyzed using Progenesis QI software. Metabolites were annotated using public databases including HMDB and LIPID MAPS. Peak alignment excluded internal standards and isotopes, with noise threshold set to 10.0. Peaks missing in more than 50% of samples were excluded from further analysis. Data were log₁₀-transformed prior to statistical testing.

Differential metabolites were identified based on a combination of variable importance in projection (VIP) scores and *P*-values from one-way ANOVA test.

**References**

**1.** Subramanian A, Tamayo P, Mootha VK, et al. Gene set enrichment analysis: A knowledge-based approach for interpreting genome-wide expression profiles. *P Natl Acad Sci USA.* 2005; 102(43):15545-15550.

**2.** Liberzon A, Subramanian A, Pinchback R, Thorvaldsdottir H, Tamayo P, Mesirov JP. Molecular signatures database (MSigDB) 3.0. *Bioinformatics.* 2011; 27(12):1739-1740.

**3.** Shannon P, Markiel A, Ozier O, et al. Cytoscape: A software environment for integrated models of biomolecular interaction networks. *Genome Res.* 2003; 13(11):2498-2504.

**4.** Love MI, Huber W, Anders S. Moderated estimation of fold change and dispersion for RNA-seq data with DESeq2. *Genome Biol.* 2014; 15(12).

**5.** Wang LB, Karpova A, Gritsenko MA, et al. Proteogenomic and metabolomic characterization of human glioblastoma. *Cancer Cell.* 2021; 39(4):509-528 e520.

**6.** Mermel CH, Schumacher SE, Hill B, Meyerson ML, Beroukhim R, Getz G. GISTIC2.0 facilitates sensitive and confident localization of the targets of focal somatic copy-number alteration in human cancers. *Genome Biol.* 2011; 12(4).

**7.** Yu K, Hu YQ, Wu F, et al. Surveying brain tumor heterogeneity by single-cell RNA-sequencing of multi-sector biopsies. *Natl Sci Rev.* 2020; 7(8):1306-1318.

**8.** Dobin A, Davis CA, Schlesinger F, et al. STAR: ultrafast universal RNA-seq aligner. *Bioinformatics.* 2013; 29(1):15-21.

**9.** Li B, Dewey CN. RSEM: accurate transcript quantification from RNA-Seq data with or without a reference genome. *Bmc Bioinformatics.* 2011; 12.
